# Estimated glucose disposal rate and cardiovascular disease risk: a meta-analysis of cohort studies

**DOI:** 10.3389/fendo.2025.1740472

**Published:** 2025-12-12

**Authors:** Zhijun Zhang, Beibei Wang, Lijuan Qu, Boping Huang

**Affiliations:** 1 Department of Cardiology, Shanxi Bethune Hospital, Shanxi Academy of Medical Sciences, Tongji Shanxi Hospital, Third Hospital of Shanxi Medical University, Taiyuan, Shanxi, China; 2 Department of Cardiology, The First People’s Hospital of Jinzhong, Jinzhong, Shanxi, China

**Keywords:** estimated glucose disposal rate, cardiovascular disease, coronary heart disease, stroke, meta-analysis, cohort studies

## Abstract

**Background:**

Insulin resistance (IR) is a key cardiovascular disease (CVD) risk factor. The estimated glucose disposal rate (eGDR) is a reliable IR marker linked to CVD risk. This study is the first extensive meta-analysis of this correlation in a general population free from baseline CVD.

**Methods:**

We searched electronic databases such as PubMed, Web of Science and Embase for cohort studies reporting eGDR and CVD risk. Studies included adults without baseline CVD, measured eGDR at baseline, and reported hazard ratio (HR) [95% confidence interval (CI)]. The combined HR and its 95% CI were determined through the application of random or fixed effects models. Meta-regression with robust error was utilized to depict the nonlinear dose-response relationship.

**Results:**

Twelve cohort studies with 547,287 subjects were included, with follow-up durations ranging from 5.6 to 14.1 years. Participants with the highest eGDR category had a lower risk of CVD (HR: 0.58, 95% CI 0.53–0.63), stroke (HR: 0.62, 95% CI: 0.56–0.69), and coronary heart disease (HR: 0.46, 95% CI: 0.25–0.83) compared with the lowest eGDR category. This aligns with the meta-analysis results, where eGDR as a continuous variable had HRs of 0.88 (95% CI: 0.85–0.91) for CVD, 0.84 (95% CI: 0.76–0.93) for stroke, and 0.85 (95% CI: 0.83–0.87) for coronary heart disease. Subgroup analyses revealed that sex, sample size, follow-up duration, and prediabetes/diabetes status did not significantly affect the results. Dose–response analysis indicated that there was a linear negative association of the eGDR with the risk of CVD (P_nonlinear_=0.120) or stroke (P_nonlinear_=0.084).

**Conclusions:**

The higher eGDR is associated with lower risk of CVD, stroke, and coronary heart disease in individuals without baseline CVD. However, the observational design and high heterogeneity across studies prevent causal inference.

## Introduction

Globally, cardiovascular diseases (CVD) are still the primary contributor to mortality and morbidity (1), with insulin resistance (IR) being a significant underlying factor (2). IR is defined by a lowered capacity of tissues to react to insulin, and it is strongly connected to metabolic syndrome. This condition is also typically linked to a Western living pattern that includes calorie-dense foods, a lack of physical activity, and persistent stress (3). This condition can lead to hyperglycemia and hyperinsulinemia, which in turn disrupt glucose metabolism and trigger a cascade of adverse health effects. These factors—such as impaired adipose tissue function, dyslipidemia, inflammation, obesity, increased reactive oxygen species (ROS) generation, endothelial dysfunction, hypertension, and atherosclerosis—are all strongly linked to the development of CVD (4, 5). The estimated glucose disposal rate (eGDR), a composite index obtained from anthropometric and laboratory data, has emerged as a reliable surrogate marker for IR. Recent studies have demonstrated that eGDR is independently linked to the risk of CVD, coronary heart disease (CHD), and stroke in diabetes or prediabetes (6). However, the consistency of this relationship across diverse cohorts and its potential as a predictive tool warrant further investigation.

Given the multifactorial nature of IR and its profound impact on cardiovascular health, understanding the correlation between eGDR and CVD risk is crucial. Prior meta-analyses have explored the role of eGDR in predicting cardiovascular risk. For example, Lei Guo et al. (7) found that higher eGDR was associated with a lower risk of CVD events in general and diabetes populations. Parham Dastjerdi et al. (8) reported similar findings in type 1 diabetes patients. Diar Zooravar et al. (9) highlighted eGDR’s potential in predicting microvascular complications in type 1 diabetes. Despite these valuable insights, prior research has not specifically examined eGDR’s association with incident CVD in populations free from baseline CVD, limiting its applicability to primary prevention. This meta-analysis advances prior knowledge by exclusively focusing on CVD-free participants to isolate true primary prevention effects, restricting inclusion to prospective cohort studies to establish temporality and minimize recall bias, and incorporating dose–response modeling to precisely quantify the shape and magnitude of the association. These enhancements provide more robust evidence regarding eGDR’s predictive utility and its continuous relationship with CVD risk.

## Methods

### Literature search

**Figure 1:**
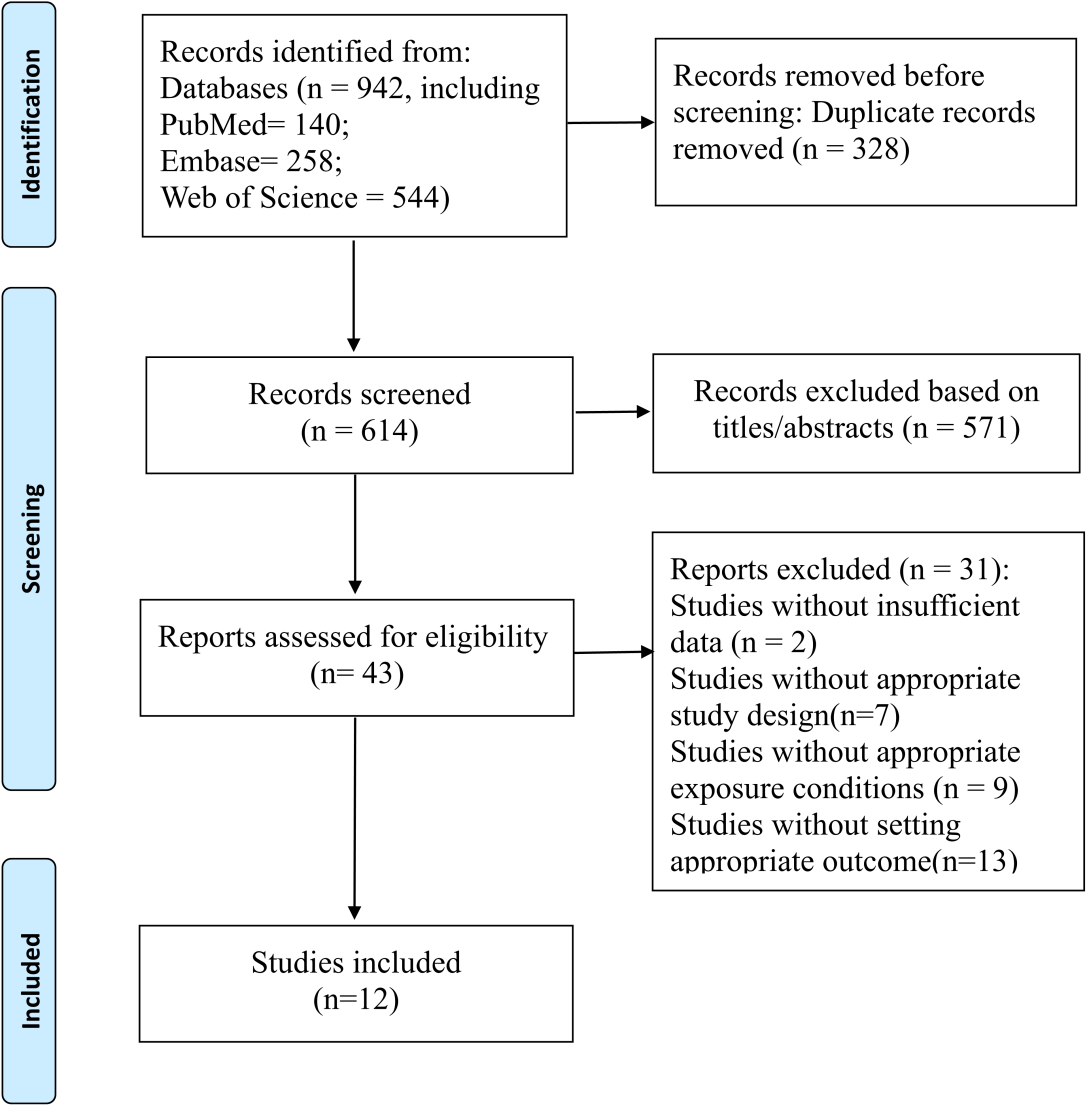
Flowchart of the database search and study identification.

This study was conducted according to the Meta-analysis of Observational Studies in Epidemiology (MOOSE) Statement (10) and PRISMA 2009 statement (11). The study selection process is shown in the PRISMA (Preferred Reporting Items for Systematic Reviews and Meta-Analyses) flow diagram (**Figure 1**). Electronic databases such as PubMed, Web of Science and Embase were searched in accordance with combined terms: (1) “estimated glucose disposal rate” OR “eGDR” and (2) “cardiovascular” OR “peripheral arterial disease” OR “coronary artery disease” OR “stroke” OR “cardiovascular disease” OR “coronary heart disease” OR” ischemic stroke” OR “CHD” OR “CVD” OR “CAD” OR “PAD” OR “IS”. The search strategy used filters to select studies involving humans and available in English. The search for final literature concluded on March 28, 2025, and the specific strategy is detailed in the **Supplementary material Table 1**. The protocol for this study has been registered in the International Prospective Register of Systematic Reviews (PROSPERO 2025 CRD 420251147324. Available from https://www.crd.york.ac.uk/PROSPERO/view/CRD420251147324).

### Study selection

The meta-analysis included studies based on these criteria: (1) The studies were cohort studies published in full. (2) eGDR was assessed at the start. (3) Participants were adults without baseline CVD. (4) Outcomes included new cases of CVD, CHD, or stroke. (5) hazard ratio (HR) [95% confidence interval (CI)] values were given. (6) The studies adjusted risk factors for possible confounders. Studies were excluded if: (1) Participants were under 18. (2) The population had baseline CVD. (3) eGDR wasn’t measured. (4) The study wasn’t a cohort design. (5) HR (95%CI) values weren’t reported.

The eGDR was calculated as (mg/kg/min) 21.158-(0.09*WC) - (3.407*HT) - (0.551*HbA1c) [WC = waist circumference (cm), HT = hypertension (yes = 1/no = 0), and HbA1c = HbA1c (% DCCT)] (12). Hypertension was defined as systolic blood pressure  ≥ 140mmHg and/or diastolic blood pressure  ≥ 90mmHg, self-reported history of hypertension, or current use of prescribed medicine for HT. HbA1c was measured by the high-performance liquid chromatography method. Furthermore, the unit in mmol/mol was transformed to a percentage (%) using the equation: (0.09148 × HbA1c mmol/mol) + 2.152 (13). CVD was identified based on self-reported physician’s diagnosis or the International Classification of Diseases (ICD-10). The main outcome was CVD, either alone or within a composite. Secondary outcomes were CHD and stroke. CAD involved chronic ischemic heart disease, angina and acute myocardial infarction. Stroke cases included both ischemic and hemorrhagic types.

Articles from Embase, Web of Science and PubMed were transferred to EndNote X9. Duplicates were identified and removed using the “duplicate identifier” function. Titles and abstracts were initially screened and categorized as potentially eligible, uncertain eligibility, or clearly ineligible. For potentially eligible or uncertain articles, full - text reviews were conducted to assess their final eligibility against the inclusion and exclusion criteria.

### Data extraction

Two researchers (Zhijun Zhang and Beibei Wang) separately performed information extraction from the articles. The data extracted included the following: (1) publication year, the first author’s name, and country; (2) the study design/mean follow-up time; (3) participant characteristics, such as the mean age, sample size, proportion of participants with prediabetes and diabetes, and proportion of male participants; (4) the analysis model used for the eGDR index; (5) the reported endpoint outcomes; and (6) the covariates controlled for within the multivariate analysis. Following the extraction process, the investigators cross-checked the data to verify its accuracy. Any discrepancies were resolved by consulting a third researcher (Lijuan Qu), whose judgment was accepted as the final decision.

### Quality evaluation

The quality of each study was assessed using the Newcastle–Ottawa Scale (14). This scale evaluates the quality of cohort studies in three aspects: study selection, comparability between groups, and outcome assessment, with scores ranging from 1 to 9 points. Studies with a score of over 6 points were regarded as high-quality.

### Data analyses

The hazard ratio (HR) and 95% confidence interval (CI) were utilized as a general measure to indicate the association between the eGDR and CVD risk in individuals without baseline CVD. For studies with the eGDR analyzed as a continuous variable, the HR (95% CI) of CVD risk per 1-unit increment of the eGDR was extracted. For studies that categorized the eGDR, the HR (95% CI) for CVD risk comparing individuals with the highest levels to those with the lowest levels of the eGDR was extracted. The heterogeneity among the included cohort studies was evaluated using the I² statistic (15). If the I² value exceeded 50%, it indicated significant heterogeneity and a random effects model was employed to pool the HR (95% CI) data; otherwise, a fixed-effects model was used for analysis. Moreover, the robustness of the results was assessed through sensitivity analyses executed by excluding each study once at a time (16). Predefined subgroup analyses were conducted to assess how study characteristics such as males sex (%), prediabetes/diabetes state (%), sample size, and mean follow-up time might influence the correlation between the eGDR and CVD risk. The potential for publication bias was initially evaluated by visually examining the symmetry of funnel plots (17). Subsequently, the trim-and-fill method, along with Egger’s (18) and Begg’s (19) tests, was employed as quantitative methods to further assess publication bias.

We computed the linear trends and 95% CI by applying the natural logarithm of the effect sizes and the 95% CI for the eGDR categories, in accordance with the method described by Greenland and Longnecker (20). Nonlinear dose - response analyses were performed via robust error meta-regression., as per the approach described by Ma and Xu et al. (21, 22) The sample fitting process was conducted in two stages. Initially, a dose–response analysis was conducted separately for each study. Subsequently, the dose–response data from these individual studies were integrated using a random-effects model. This model necessitates information on the known levels of eGDR, the natural logarithm of the HR, the number of cases and the person-year (calculated by multiplying the average follow-up time by the number of cases) within each exposure range (22). When quantitative eGDR values were unavailable, missing values were imputed using the method detailed by Xu et al. (21). This approach allows for the use of either the exposure median or mean. In cases where neither the mean nor median is provided but a range of values is reported, the exposure level can be approximated as follows: for closed intervals, the midpoint between the upper and lower bounds is used; for open intervals, the interval length is inferred from the adjacent group, and the midpoint of this interval is taken as the average exposure level (23). The meta-analysis and statistical analysis were performed using R (4.2.2) software. A p-value less than 0.05 or 95% CI excluding 1 was regarded as statistically significant.

## Results

### Literature search

As depicted in **Figure 1**, the database search process was conducted systematically. A total of 614 articles were retrieved from the initial search of Web of Science, PubMed and Embase databases, following the removal of duplicate entries. During the preliminary screening of titles and abstracts, 571 articles were deemed irrelevant and excluded. Subsequently, 31 articles were further excluded based on the criteria outlined in **Supplementary material Table 2**. In the end, twelve cohort studies (24–35) were finalized for inclusion in the meta-analysis.

### Study characteristics

The characteristics of the twelve cohort studies are shown in **Supplementary material Table 3**. Twelve cohort studies with 547,287 subjects were included, with follow-up durations ranging from 5.6 to 14.1 years. These studies were carried out in China, Sweden, England, and the United States of America. All studies were cohort studies and published between 2022 and 2024.The average age of the participants across the twelve studies spanned from 56.3 to 62.9 years. Two studies had a male participant proportion exceeding 50%, while the remaining studies featured a male participant proportion under 50%. Additionally, three articles featured sample sizes exceeding 10,000, whereas nine articles reported sample sizes below 10,000.

### Quality evaluation

This meta-analysis included twelve cohort studies. Their quality was evaluated via the Newcastle–Ottawa Scale, where the highest possible score is 8. The assessment showed that three studies achieved a score of 7, while the remaining seven studies scored 8. Thus, all included cohort studies were deemed high-quality (**Supplementary material Table 4**).

### eGDR and CVD risk

**Figure 2:**
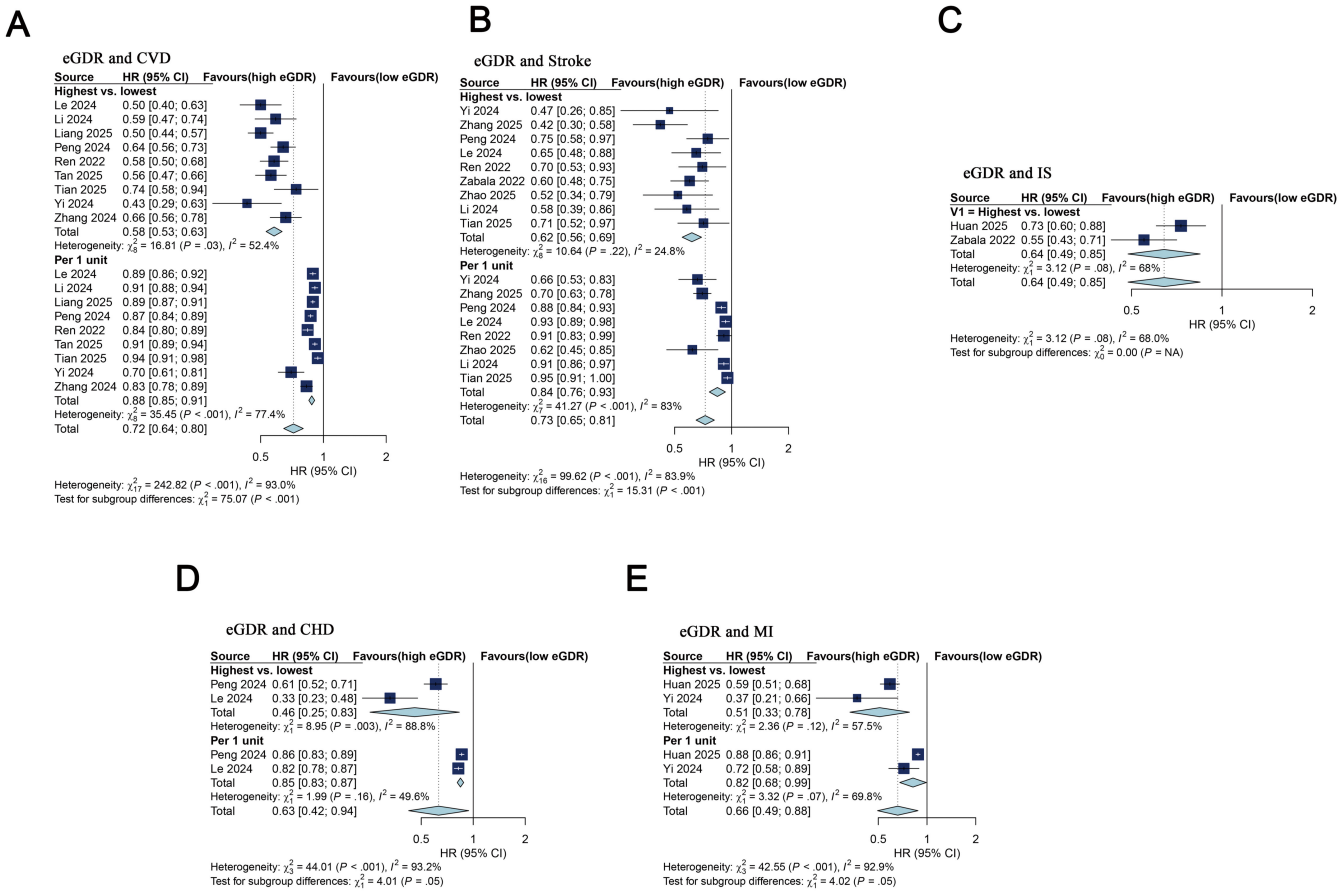
Forest plot for meta-analysis. **(A)** Association between eGDR (mg/kg/min) and CVD risk. Pooled HR: 0.58 (95% CI: 0.53–0.63) for highest vs. lowest eGDR; 0.88 (95% CI: 0.85–0.91) per 1-unit. Random-effects models used (I² = 52.4% for highest vs. lowest eGDR and 77.4% for per 1-unit). **(B)** Association between eGDR (mg/kg/min) and stroke risk. Pooled HR: 0.62 (95% CI 0.56–0.69) for highest vs. lowest eGDR, 0.73 (95% CI 0.65–0.81) for per 1-unit. Models: Random-effects (I² = 83%) for per 1-unit; fixed-effects (I² = 24.8%) for highest vs. lowest eGDR. **(C)** Association between eGDR (mg/kg/min) and IS risk: Pooled HR: 0.64 (95% CI 0.49–0.85) for highest vs. lowest eGDR. Random-effects model (I² = 68%). **(D)** Association between eGDR and CHD risk: Pooled HR: 0.46 (95% CI 0.25–0.83) for highest vs. lowest eGDR, 0.85 (95% CI 0.83–0.87) for per 1-unit). Models: Random-effects (I² = 88.8%) for highest vs. lowest eGDR; fixed-effects (I² = 49.6%) for per 1-unit. **(E)** Association between eGDR and MI risk: Pooled HR: 0.51 (95% CI 0.33–0.78) for highest vs. lowest eGDR, 0.82(95% CI 0.68–0.99) for per 1-unit. Random-effects models used (I² = 57.5% for highest vs. lowest eGDR and 69.8% for per 1-unit). HR, hazard ratio; CI, confidence interval; eGDR, estimated glucose disposal rate; CVD, cardiovascular disease; CHD, coronary heart disease; MI, myocardial infarction; IS, ischemic stroke.

**Figure 3:**
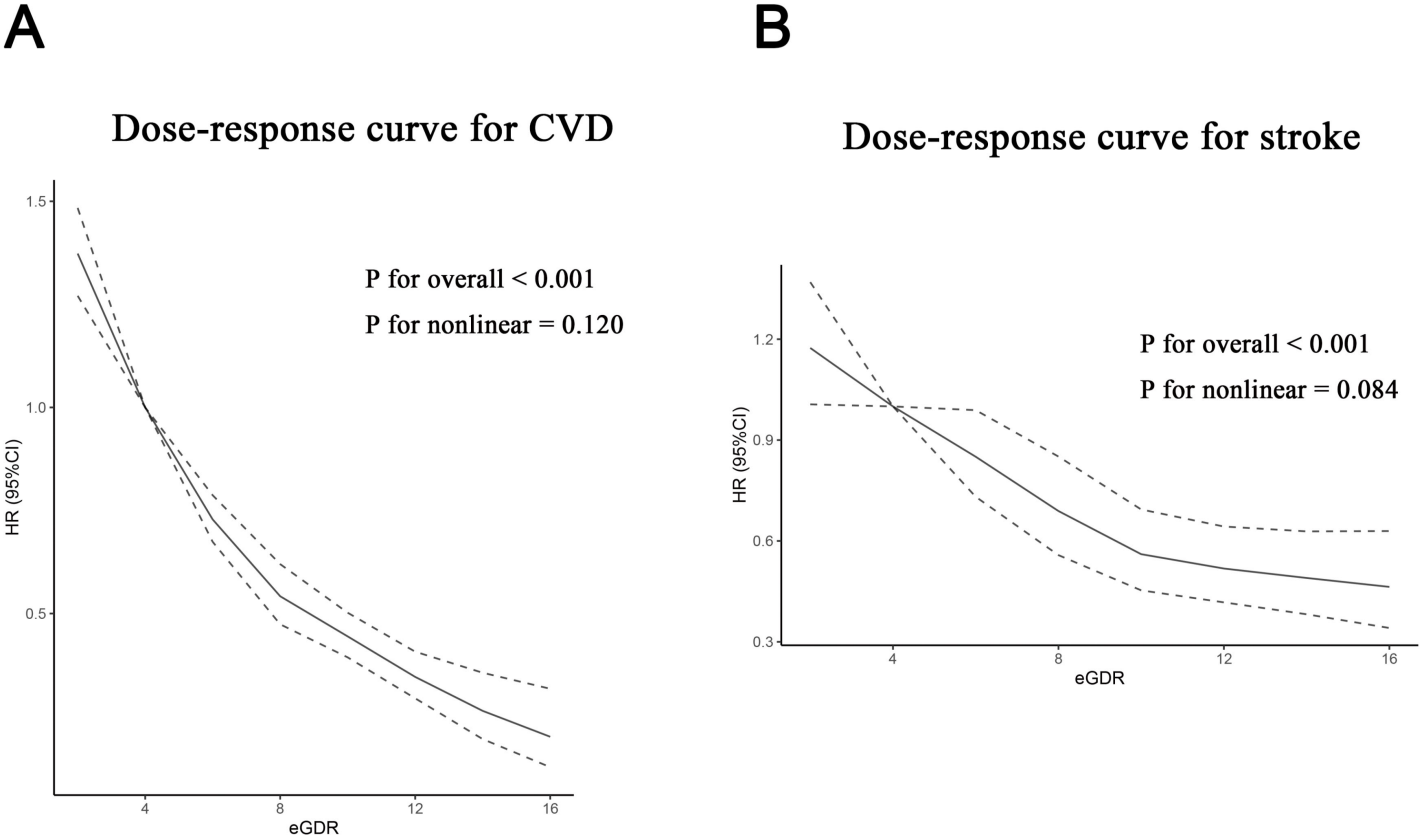
Dose-response curves for the association between eGDR (mg/kg/min) and CVD risk were generated when eGDR was analyzed as a categorical variable. The x-axis displays eGDR values ranging from 0 to 16.0 mg/kg/min, and the y-axis represents the estimated hazard ratio. The solid line represents the estimated hazard ratio, and the dashed lines represent the 95% confidence interval for this continuous exposure model. **(A)** Association with CVD risk. The relationship is linear (P for nonlinearity = 0.120). **(B)** Association with stroke risk. The relationship is linear (P for nonlinearity = 0.084). Abbreviations: eGDR, estimated glucose disposal rate; CVD, cardiovascular disease.

For CVD analysis, a total of nine cohorts (25–29, 31, 33–35)were examined, covering 73,577 participants. A random-effects model was used, with eGDR being treated as a categorical variable. The pooled results from the nine cohorts demonstrated that participants in the highest eGDR category experienced a significantly reduced CVD risk compared to those in the lowest eGDR category at baseline (HR = 0.58; 95% CI 0.53–0.63; I^2^ = 52.4%; **Figure 2A**). This matched the meta-analysis of eGDR as a continuous variable, with a 12% (HR: 0.88, 95% CI 0.85–0.91, I²=77.4%) reduction in CVD risk for every 1-unit increase (**Figure 2A**). Dose-response curves treating eGDR as a categorical variable showed a negative linear relationship was observed between the eGDR and CVD risk (P_nonlinear_ = 0.120) (**Figure 3A**). **Supplementary material Table 5** presents estimates for the linear exposure effect analysis for eGDR.

In subgroup evaluations, those in the highest eGDR group showed a significantly lower CVD risk than the lowest group, and this finding was consistent regardless of male sex (%), prediabetes/diabetes statue (%), sample size, and mean follow-up time (P > 0.05 for each subgroup; **Table 1**). Omitting one study at a time in sensitivity analyses generated analogous results (HR for the eGDR analyzed as a categorical variable: 0.57–0.60, all P<0.05) (**Supplementary material Figure 1A**). Funnel plots exhibited symmetrical features on visual inspection when eGDR was evaluated as a categorical variable, implying a low propensity for publication bias (**Supplementary Figure 1B**). The P values were 0.750 and 0.835 based on Begg’s and Egger’s regression, respectively, further suggesting no publication bias (**Supplementary material Figures 2A, B**). After incorporating one study using the trim-and-fill method, the HR (95% CI) remained largely unchanged, indicating that the combined effect size results are reliable (**Supplementary material Figures 3A, B**).

**Table 1:** Subgroup analyses for the association between the estimated glucose disposal rate analyzed as a categorical variable and the risk of cardiovascular disease.

Subgrouped by	Number of studies	Effect value HR (95% CI)	I-squared (%)	P interaction
Male%	9	0.58 [0.53;0.63]	52.4%	0.58
≥50%	3	0.62 [0.49;0.78]	80.9%	
<50%	6	0.57 [0.53;0.63]	20.5%	
Follow up	9	0.58 [0.53;0.63]	52.4%	0.07
>7 years	6	0.55 [0.49;0.61]	46.4%	
≤7 years	3	0.64 [0.56;0.73]	33.8%	
Sample size	9	0.58[0.53;0.63]	52.4%	0.78
≥7,000	3	0.57[0.49;0.66]	50.4%	
<7,000	6	0.59[0.52;0.67]	68.5%	
Pre-DM/DM	7	0.58[0.52;0.64]	58.4%	0.99
≥20%	2	0.58[0.34;0.98]	82.2%	
<20%	5	0.57[0.52;0.64]	52.1%	

Pre-DM, pre- diabetes mellitus; DM, diabetes mellitus; HR, hazard ratio; CI, confidence interval.

### eGDR and stroke risk

Nine cohorts (25–30, 32–34)were included in the stroke analysis. The combined findings showed that higher eGDR corresponded to a lower stroke risk when comparing the highest and lowest categories (HR = 0.62; 95% CI 0.56–0.69; I²=24.8%) (**Figure 2B**). These findings were aligned with the meta-analysis of eGDR as a continuous variable, with a 16% (HR: 0.84, 95% CI 0.76–0.93, I²=83.0%) reduction in stroke risk for every 1-unit increase (**Figure 2B**). Dose-response analysis treating eGDR as a categorical variable showed a negative linear correlation between eGDR and stroke risk (P_nonlinear_=0.084) (**Figure 3B**). **Supplementary material Table 5** presents HR(95%CI) estimates for the linear exposure effect analysis for the eGDR. Omitting one study at a time in sensitivity analyses generated analogous results (HR for the eGDR analyzed as a categorical variable: 0.59–0.65, all P<0.05) (**Supplementary material Figure 1C**). When eGDR was analyzed as a categorical variable, the funnel plots appeared symmetric upon visual assessment, indicating a minimal risk of publication bias. (**Supplementary material Figure 1D**). The P values were 0.061 and 0.181 based on Begg’s and Egger's regression, respectively (**Supplementary material Figures 2C, D**). The trim-and-fill method was used to add three studies, and the HR (95% CI) did not change significantly, indicating that the combined effect size results were robust (**Supplementary material Figures 3C, D**).

Two cohorts (24, 30)were included in the ischemic stroke analysis. The aggregated data showed that participants in the highest eGDR group exhibited a decreased risk of ischemic stroke risk (HR = 0.64; 95% CI 0.49–0.85; I²=68%) when compared with those in the lowest eGDR group (**Figure 2C**). This results was in line with the Huan et al. (24) treating eGDR as a continuous variable, with a 13% (HR: 0.87, 95% CI 0.83–0.90) reduction in ischemic stroke risk for every 1-unit increase (**Supplementary material Table 1**).

### eGDR and CHD risk

The pooled estimates from two cohort studies (27, 28) indicated that higher eGDR was related to a lower CHD risk when treated as a categorical variable (HR = 0.46; 95% CI 0.25–0.83; I²=88.8%), and consistent results were found when eGDR was treated as a continuous variable (HR = 0.85; 95% CI 0.83–0.87; I²=49.6%) (**Figure 2D**). Similarly, higher eGDR was correlated with a reduced myocardial infarction risk in both categorical (HR = 0.51; 95% CI 0.33–0.78; I^2^ = 57.5%) and continuous analyses (HR = 0.82; 95% CI 0.68–0.99; I^2^ = 69.8%) (**Figure 2E**).

## Discussion

IR is a cornerstone in the pathogenesis of CVD, and the eGDR has become a promising alternative measure for assessing IR. The results of our meta-analysis are consistent with those of previous studies (6, 36, 37) that demonstrated the link between IR and CVD risk. The pooled HR for CVD, stroke, and CHD across different eGDR categories and continuous eGDR further strengthen the notion that better insulin sensitivity, as indicated by higher eGDR, is protective against CVD events. We conducted sensitivity analyses by excluding each study one at a time, which consistently yielded similar results, thereby confirming the stability of our findings. Additionally, dose–response analyses revealed a linear trend, supporting the idea that even small improvements in eGDR could translate into meaningful reductions in CVD risk. Subgroup analyses across various factors such as sex, sample size, follow-up duration, and prediabetes/diabetes status also demonstrated consistent results, further strengthening the credibility and generalizability of our conclusions. This finding underscores the potential of eGDR as a predictive marker for CVD risk and highlights its clinical and public health significance.

While insulin resistance indices such as homeostatic model assessment of insulin resistance (HOMA-IR) and the triglyceride Glucose (TyG) Index have demonstrated associations with CVD risk (38–41), eGDR offers distinct practical advantages for CVD risk assessment. HOMA-IR requires fasting insulin measurements, which are not routinely available in clinical practice and may be unreliable in patients receiving insulin or insulin-sensitizing medications (42). Furthermore, HOMA-IR primarily reflects hepatic insulin resistance and may not capture peripheral insulin sensitivity as comprehensively. The TyG index, though more accessible as it uses fasting glucose and triglycerides, has shown inconsistent predictive performance across populations and may be less reliable in non-fasting states or in individuals with significant hypertriglyceridemia. In contrast, eGDR is calculated from three routinely measured clinical parameters—waist circumference, hypertension status, and HbA1c—facilitating its application in large-scale risk stratification. This composite approach enables eGDR to capture both metabolic and hemodynamic components of IS simultaneously. Notably, eGDR demonstrates comparable accuracy to the hyperinsulinemic-euglycemic clamp—the gold standard for insulin resistance assessment—while avoiding its invasive nature and substantial cost (2, 43, 44). The inclusion of waist circumference and hypertension status may also explain eGDR’s strong association with cardiovascular outcomes, as these factors independently predict CVD risk. This combination of comprehensive risk capture, practical utility, and strong correlation with outcomes underscores eGDR’s value as a predictive marker for cardiovascular risk in routine clinical settings.

IR contributes to CVD through several mechanisms. First, IR results in heightened free fatty acid concentrations in the bloodstream, which can accumulate and exert toxic effects on the cardiovascular system (2, 45). Second, IR is related to various inflammatory markers, such as monocyte chemoattractant protein-1, leptin, tumor necrosis factor-alpha, plasminogen activator inhibitor-1, interleukin-6 and adiponectin (2, 5). These markers promote the development of atherosclerosis. Third, IR is frequently correlated with abnormal lipid profiles, such as small dense low-density lipoprotein cholesterol (LDL-C), elevated LDL-C, heightened hepatic triglycerides, and reduced high-density LDL-C (2, 5, 43). Fourthly, in the state of IR, the production of nitrogen species and reactive oxygen species increases, leading to oxidative stress. These effects damage endothelial cells and promote atherosclerosis (2, 44, 45). Fifth, insulin helps maintain endothelial function by regulating nitric oxide (NO) production. In IR, NO production is reduced, impairing vasodilation and contributing to hypertension and atherosclerosis. (2, 44, 45) Sixth, IR is closely linked to high blood pressure through mechanisms such as overactivation of the renin-angiotensin-aldosterone system, causing vasoconstriction and sodium retention, as well as increased sympathetic nervous system activity, leading to myocardial hypertrophy, interstitial fibrosis, and reduced contractile function (2). Finally, IR causes myocardial metabolic disturbances, characterized by increased fatty acid oxidation and decreased glucose oxidation. This metabolic imbalance results in insufficient myocardial energy production, affecting cardiac contraction and relaxation (43).

### Strengths and limitations

As far as we are aware, this is the first meta-analysis to explore the association between eGDR and CVD risk in a general population that was free from CVD at baseline, which allows for a more accurate assessment of the predictive value of eGDR in individuals without pre-existing CVD. By exclusively including cohort studies, we circumvented potential recall bias inherent in cross-sectional designs, thereby strengthening the causal inference in the observed association. Moreover, we conducted separate meta-analyses treating eGDR as both a categorical and a continuous variable, a methodological approach that provided complementary perspectives and further validated the robustness of our primary conclusions. Despite the presence of significant heterogeneity, we conducted sensitivity, dose–response, and subgroup analyses, which yielded robust and reliable results. Additionally, cohort studies released in the past three years, all of our selected studies were of high quality and they featured extensive sample sizes along with lengthy follow-up intervals.

Although this meta-analysis has notable strengths and potential clinical relevance, several limitations exist that warrant consideration when evaluating the results. First, despite the use of random-effects models to account for heterogeneity, significant heterogeneity was still observed in some analyses. This heterogeneity may stem from differences in participants’ races and comorbidities, as well as methods for measuring eGDR and CVD outcomes. Second, the potential for residual confounding, such as dietary patterns (46, 47), sleep quality and sleep duration factors (48), and liver fibrosis (49) may have an impact on eGDR, in the original cohort studies cannot be fully excluded, although most studies adjusted for multiple potential confounders. Third, most studies were from China and only three from Sweden/USA, limiting ethnic diversity and generalizability to global cardiovascular risk patterns. Future research must validate eGDR cutoffs and predictive accuracy in diverse Western and multi-ethnic cohorts. Fourth, it is important to note that the observational nature of the included studies limits our ability to establish causality between eGDR and cardiovascular outcomes. While the findings suggest a strong association, confounding factors and reverse causation cannot be entirely ruled out. Finally, eGDR formula was originally validated using clamp studies in type 1 diabetes populations, and its metabolic validity in different populations has not been directly established. However, this meta-analysis aims to evaluate predictive validity rather than metabolic validity, and the potential non-differential misclassification bias is more likely to make our risk estimation conservative than exaggerated.

### Conclusions

Our meta-analysis reveals that higher eGDR is associated with a significantly lower risk of CVD, stroke, and CHD. This indicates that eGDR could serve as a valuable marker for predicting CVD risk in individuals without baseline CVD. Future research should focus on further exploring the underlying mechanisms and assessing the predictive power of eGDR in diverse populations.

## Data Availability

The original contributions presented in the study are included in the article/**Supplementary Material**. Further inquiries can be directed to the corresponding author.

## References

[1] GBD-NHLBI-JACC Global Burden of Cardiovascular Diseases Writing Group . Global burden of cardiovascular diseases and risk factors, 1990-2019: update from the GBD 2019 study. J Am Coll Cardiol. (2020) 76:2982–3021. doi: 10.1016/j.jacc.2020.11.010, PMID: 33309175 PMC7755038

[2] Kosmas CE Bousvarou MD Kostara CE Papakonstantinou EJ Salamou E Guzman E . Insulin resistance and cardiovascular disease. J Int Med Res. (2023) 51:3000605231164548. doi: 10.1177/03000605231164548, PMID: 36994866 PMC10069006

[3] Saklayen MG . The global epidemic of the metabolic syndrome. Curr Hypertens Rep. (2018) 20:12. doi: 10.1007/s11906-018-0812-z, PMID: 29480368 PMC5866840

[4] DeFronzo RA . Insulin resistance, lipotoxicity, type 2 diabetes and atherosclerosis: the missing links. The Claude Bernard Lecture 2009. Diabetologia. (2010) 53:1270–87. doi: 10.1007/s00125-010-1684-1, PMID: 20361178 PMC2877338

[5] Yaribeygi H Farrokhi FR Butler AE Sahebkar A . Insulin resistance: Review of the underlying molecular mechanisms. J Cell Physiol. (2019) 234:8152–61. doi: 10.1002/jcp.27603, PMID: 30317615

[6] Liao J Wang L Duan L Gong F Zhu H Pan H . Association between estimated glucose disposal rate and cardiovascular diseases in patients with diabetes or prediabetes: a cross-sectional study. Cardiovasc Diabetol. (2025) 24:13. doi: 10.1186/s12933-024-02570-y, PMID: 39806389 PMC11730478

[7] Guo L Zhang J An R Wang W Fen J Wu Y . The role of estimated glucose disposal rate in predicting cardiovascular risk among general and diabetes mellitus population: a systematic review and meta-analysis. BMC Med. (2025) 23:234. doi: 10.1186/s12916-025-04064-4, PMID: 40264086 PMC12016375

[8] Dastjerdi P Mohammadi NSH Anaraki N Rahmati S Nikfar R Momeni S . Estimated glucose disposal rate and risk of cardiovascular events in type 1 diabetes: a systematic review and meta-analysis. Diabetol Metab Syndrome. (2025) 17:348. doi: 10.1186/s13098-025-01900-8, PMID: 40841970 PMC12369242

[9] Zooravar D Radkhah H Amiri BS Soltani P . Estimated glucose disposal rate and microvascular complications of diabetes mellitus type I: A systematic review and meta-analysis. Diabetes Vasc Dis Res. (2025) 22:1479164125 132–4612. doi: 10.1177/14791641251324612, PMID: 40114403 PMC11926832

[10] Stroup DF Berlin JA Morton SC Olkin I Williamson GD Rennie D . Meta-analysis of observational studies in epidemiology: a proposal for reporting. Meta-analysis Of Observational Studies in Epidemiology (MOOSE) group. JAMA. (2000) 283:2008–12. doi: 10.1001/jama.283.15.2008, PMID: 10789670

[11] Moher D Liberati A Tetzlaff J Altman DG The PRISMA Group . Preferred reporting items for systematic reviews and meta-analyses: the PRISMA statement. PloS Med. (2009) 6:e1000097. doi: 10.1371/journal.pmed.1000097, PMID: 19621072 PMC2707599

[12] Epstein EJ Osman JL Cohen HW Rajpathak SN Lewis O Crandall JP . Use of the estimated glucose disposal rate as a measure of insulin resistance in an urban multiethnic population with type 1 diabetes. Diabetes Care. (2013) 36:2280–5. doi: 10.2337/dc12-1693, PMID: 23596179 PMC3714518

[13] English E Lenters-Westra E . HbA1c method performance: The great success story of global standardization. Crit Rev Clin Lab Sci. (2018) 55:408–19. doi: 10.1080/10408363.2018.1480591, PMID: 30001673

[14] The Newcastle-Ottawa Scale . (NOS) for assessing the quality of non-ran domised studies in meta-analyses (2010). Available online at: https://www.ohri.ca/programs/clinical_epidemiology/oxford.asp (Accessed April 22, 2025).

[15] Bowden J Tierney JF Copas AJ Burdett S . Quantifying, displaying and accounting for heterogeneity in the meta-analysis of RCTs using standard and generalised Q statistics. BMC Med Res Methodol. (2011) 11:41. doi: 10.1186/1471-2288-11-41, PMID: 21473747 PMC3102034

[16] Patsopoulos NA Evangelou E Ioannidis JP . Sensitivity of between-study heterogeneity in meta-analysis: proposed metrics and empirical evaluation. Int J Epidemiol. (2008) 37:1148–57. doi: 10.1093/ije/dyn065, PMID: 18424475 PMC6281381

[17] Zwetsloot PP van der Naald M Sena ES Howells DW IntHout J De Groot JA . Standardized mean differences cause funnel plot distortion in publication bias assessments. Elife. (2017) :6:e24260. doi: 10.7554/eLife.24260, PMID: 28884685 PMC5621838

[18] Egger M Davey Smith G Schneider M Minder C . Bias in meta-analysis detected by a simple, graphical test. BMJ (Clinical Res ed.). (1997) 315:629–34. doi: 10.1136/bmj.315.7109.629, PMID: 9310563 PMC2127453

[19] Begg CB Mazumdar M . Operating characteristics of a rank correlation test for publication bias. Biometrics. (1994) 50:1088–101. doi: 10.2307/2533446, PMID: 7786990

[20] Greenland S Longnecker MP . Methods for trend estimation from summarized dose-response data, with applications to meta-analysis. Am J Epidemiol. (1992) 135:1301–9. doi: 10.1093/oxfordjournals.aje.a116237, PMID: 1626547

[21] Xu C Doi SAR . The robust error meta-regression method for dose-response meta-analysis. Int J Evidence-Based Healthcare. (2018) 16:138–44. doi: 10.1097/XEB.0000000000000132, PMID: 29251651

[22] Ma X Yang Y Wang Y Zhao L Sun J Tan Y . Statistical method and application of dose-response analyses in Meta-analysis. Fudan Univ J Med Sci. (2015) 42:123–8. doi: 10.3969/j.issn.1672-8467.2015.01.023

[23] Xu C LT Kuang X Zhang Y Weng H Zhnag C . How to estimate the missing data and transform the effect measure in dose-response meta-analysis. Methodology. (2015) 15:984–7. doi: 10.7507/1672-2531.20150164

[24] Huang H Xiong Y Zhou J Tang Y Chen F Li G . The predictive value of estimated glucose disposal rate and its association with myocardial infarction, heart failure, atrial fibrillation and ischemic stroke. Diabetes Obes Metab. (2025) 3:1359-68. doi: 10.1111/dom.16132, PMID: 39743837

[25] Yi J Qu C Li X Gao H . Insulin resistance assessed by estimated glucose disposal rate and risk of atherosclerotic cardiovascular diseases incidence: the multi-ethnic study of atherosclerosiss. Cardiovasc Diabetol. (2024) 23:349. doi: 10.1186/s12933-024-02437-2, PMID: 39342205 PMC11439291

[26] Zhang Z Zhao L Lu Y Xiao Y Zhou X . Insulin resistance assessed by estimated glucose disposal rate and risk of incident cardiovascular diseases among individuals without diabetes: findings from a nationwide, population based, prospective cohort study. Cardiovasc Diabetol. (2024) 23:194. doi: 10.1186/s12933-024-02256-5, PMID: 38844981 PMC11157942

[27] Peng J Zhang Y Zhu Y Chen W Chen L Ma F . Estimated glucose disposal rate for predicting cardiovascular events and mortality in patients with non-diabetic chronic kidney disease: a prospective cohort study. BMC Med. (2024) 22:411. doi: 10.1186/s12916-024-03582-x, PMID: 39334214 PMC11438365

[28] Le C Qin Y Wang Z Wang D Zhong F Yang S . Association of estimated glucose disposal rate with incident cardiovascular disease under different metabolic and circadian rhythm states: findings from a national population-based prospective cohort study. Diabetol Metab Syndrome. (2024) 16:257. doi: 10.1186/s13098-024-01494-7, PMID: 39472994 PMC11523584

[29] Ren X Jiang M Han L Zheng X . Estimated glucose disposal rate and risk of cardiovascular disease: evidence from the China Health and Retirement Longitudinal Study. BMC Geriatr. (2022) 22:968. doi: 10.1186/s12877-022-03689-x, PMID: 36517754 PMC9753298

[30] Zabala A Darsalia V Lind M Svensson A-M Franzén S Eliasson B . Estimated glucose disposal rate and risk of stroke and mortality in type 2 diabetes: a nationwide cohort study. Cardiovasc Diabetol. (2021) 20:202. doi: 10.1186/s12933-021-01394-4, PMID: 34615525 PMC8495918

[31] Liang X Lai K Li X Gui S Xing Z Li Y . U-shaped relationship of estimated glucose disposal rate with cardiovascular disease risk in cardiovascular-kidney-metabolic syndrome stages 0-3: a population-based prospective study. Diabetol Metab Syndrome. (2025) 17:85. doi: 10.1186/s13098-025-01659-y, PMID: 40069902 PMC11895221

[32] Zhao Z Liu Y Zheng J Li J . The role of glucose disposal efficiency in predicting stroke among older adults: a cohort study. Front Neurol. (2025) 16:1540160. doi: 10.3389/fneur.2025.1540160, PMID: 40134698 PMC11932850

[33] Li Y Li H Chen X Liang X . Association between various insulin resistance indices and cardiovascular disease in middle-aged and elderly individuals: evidence from two prospectives nationwide cohort surveys. Front Endocrinol. (2024) 15:1483468. doi: 10.3389/fendo.2024.1483468, PMID: 39649228 PMC11620891

[34] Tian J Chen H Luo Y Zhang Z Xiong S Liu H . Association between estimated glucose disposal rate and prediction of cardiovascular disease risk among individuals with cardiovascular-kidney-metabolic syndrome stage 0-3: a nationwide prospective cohort study. Diabetol Metab Syndrome. (2025) 17:58. doi: 10.1186/s13098-025-01626-7, PMID: 39953554 PMC11827371

[35] Tan Z Zhou D Tang Y Huo G . Association between estimated glucose disposal rate and incident cardiovascular disease in a population with Cardiovascular-Kidney-Metabolic syndrome stages 0-3: insights from CHARLS. Front Cardiovasc Med. (2025) 12:1537774. doi: 10.3389/fcvm.2025.1537774, PMID: 40066350 PMC11891229

[36] Sun R Wang J Li M Li J Pan Y Liu B . Association of insulin resistance with cardiovascular disease and all-cause mortality in type 1 diabetes: systematic review and meta-analysis. Diabetes Care. (2024) 47:2266–74. doi: 10.2337/dc24-0475, PMID: 39018337

[37] Kong X Wang W . Estimated glucose disposal rate and risk of cardiovascular disease and mortality in U.S. adults with prediabetes: a nationwide cross-sectional and prospective cohort study. Acta Diabetol. (2024) 61:1413–21. doi: 10.1007/s00592-024-02305-1, PMID: 38805079

[38] Yan F Yan S Wang J Cui Y Chen F Fang F . Association between triglyceride glucose index and risk of cerebrovascular disease: systematic review and meta-analysis. Cardiovasc Diabetol. (2022) 1:226. doi: 10.1186/s12933-022-01664-9, PMID: 36324146 PMC9632026

[39] Bonora E Kiechl S Willeit J Oberhollenzer F Egger G Meigs JB . Insulin resistance as estimated by homeostasis model assess-ment predicts incident symptomatic cardiovascular disease in caucasian subjects from the general population: the Bruneck study. Diabetes Care. (2007) 30:318–24.33., PMID: 17259501 10.2337/dc06-0919

[40] van der Aa MP Elst MA van de Garde EM van Mil EG Knibbe CA van der Vorst MM . Long-term treatment with metformin in obese, insulin-resistant adolescents: results of a randomized double-blinded placebo-controlled trial. Nutr Diabetes. (2016) 6:e228., PMID: 27571249 10.1038/nutd.2016.37 PMC5022149

[41] Liu X Tan Z Huang Y Zhao H Liu M Yu P . Relationship between the triglyceride-glucose index and risk of cardiovascular diseases and mortality in the general population: a systematic review and meta-analysis. Cardiovasc Diabetol. (2022) 1:124. doi: 10.1186/s12933-022-01546-0, PMID: 35778731 PMC9250255

[42] Wang T Li M Zeng T Hu R Xu Y Xu M . Association Between Insulin Resistance and Cardiovascular Disease Risk Varies According to Glucose Tolerance Status: A Nationwide Prospective Cohort Study. Diabetes Care. (2022) 45:1863–1872. doi: 10.2337/dc22-0202, PMID: 35700159 PMC9346991

[43] Ormazabal V Nair S Elfeky O Aguayo C Salomon C Zuñiga FA . Association between insulin resistance and the development of cardiovascular disease. Cardiovasc Diabetol. (2018) 17:122. doi: 10.1186/s12933-018-0762-4, PMID: 30170598 PMC6119242

[44] Hill MA Yang Y Zhang L Sun Z Jia G Parrish AR . Insulin resistance, cardiovascular stiffening and cardiovascular disease. Metabolism. (2021) 119:154766. doi: 10.1016/j.metabol.2021.154766, PMID: 33766485

[45] Mathew M Tay E Cusi K . Elevated plasma free fatty acids increase cardiovascular risk by inducing plasma biomarkers of endothelial activation, myeloperoxidase and PAI-1 in healthy subjects. Cardiovasc Diabetol. (2010) 9:9. doi: 10.1186/1475-2840-9-9, PMID: 20158910 PMC2837624

[46] Kietsiriroje N Shah H Zare M O’Mahoney LL West DJ Pearson SM . Dietary fat intake is associated with insulin resistance and an adverse vascular profile in patients with T1D: a pooled analysis. Eur J Nutr. (2023) 62:1231–8. doi: 10.1007/s00394-022-03070-z, PMID: 36495341 PMC10030402

[47] Shojaeian Z Ebrahimi Z Amiri F Esmaillzadeh A Sadeghi O Jahed SA . Associations of major dietary patterns with cardiometabolic risk factors among Iranian patients with type 1 diabetes. Prev Med Rep. (2024) 38:102618. doi: 10.1016/j.pmedr.2024.102618, PMID: 38375177 PMC10874838

[48] Rusu A Bala C Ciobanu D Cerghizan A Roman G . Sleep quality and sleep duration, but not circadian parameters are associated with decreased insulin sensitivity in Type 1 diabetes. Chronobiol Int. (2019) 36:1148–55. doi: 10.1080/07420528.2019.1615501, PMID: 31117834

[49] Lonardo A Ballestri S Baffy G Weiskirchen R . Liver fibrosis as a barometer of systemic health by gauging the risk of extrahepatic disease. Metab Target Organ Damage. (2024) 4:41. doi: 10.20517/mtod.2024.42

