# Supplementary material for: Estimated glucose disposal rate and cardiovascular disease risk: a meta-analysis of cohort studies
Source: Front Endocrinol (Lausanne). 2025 Dec 12;16:1740472. doi: 10.3389/fendo.2025.1740472 (PMC12740917; doi:10.3389/fendo.2025.1740472)

Table 1: Detailed description of database search strategy

Datebase	Search	Query	Result
Embase	#1	'arterial disease, peripheral occlusive'/exp OR 'arterial disease, peripheral occlusive' OR 'arterial obliterative disease'/exp OR 'arterial obliterative disease' OR 'arterial occlusive diseases'/exp OR 'arterial occlusive diseases' OR 'artery chronic occlusive disease'/exp OR 'artery chronic occlusive disease' OR 'artery obliterative disease'/exp OR 'artery obliterative disease' OR 'artery occlusive disease'/exp OR 'artery occlusive disease' OR 'chronic arterial occlusion disease'/exp OR 'chronic arterial occlusion disease' OR 'chronic artery obstruction'/exp OR 'chronic artery obstruction' OR 'chronic artery occlusion'/exp OR 'chronic artery occlusion' OR 'chronic occlusion, artery'/exp OR 'chronic occlusion, artery' OR 'obliterative arterial disease'/exp OR 'obliterative arterial disease' OR 'obliterative artery disease'/exp OR 'obliterative artery disease' OR 'obstructive arterial disease'/exp OR 'obstructive arterial disease' OR 'obstructive artery disease'/exp OR 'obstructive artery disease' OR 'occlusive arterial disease'/exp OR 'occlusive arterial disease' OR 'occlusive artery disease'/exp OR 'occlusive artery disease' OR 'pad (peripheral arterial disease)'/exp OR 'pad (peripheral arterial disease)' OR 'paod'/exp OR 'paod' OR 'peripheral arterial diseases'/exp OR 'peripheral arterial diseases' OR 'peripheral arterial disorder'/exp OR 'peripheral arterial disorder' OR 'peripheral arterial obstructive disease'/exp OR 'peripheral arterial obstructive disease' OR 'peripheral arterial occlusive disease'/exp OR 'peripheral arterial occlusive disease' OR 'peripheral arterial occlusive diseases'/exp OR 'peripheral arterial occlusive diseases' OR 'peripheral artery disease'/exp OR 'peripheral artery disease' OR 'peripheral artery obstructive disease'/exp OR 'peripheral artery obstructive disease' OR 'peripheral artery occlusive disease'/exp OR 'peripheral artery occlusive disease' OR 'peripheral atherosclerosis'/exp OR 'peripheral atherosclerosis' OR 'peripheral obliterative arterial disease'/exp OR 'peripheral obliterative arterial disease' OR 'peripheral obliterative vascular disease'/exp OR 'peripheral obliterative vascular disease' OR 'peripheral obstructive artery disease'/exp OR 'peripheral obstructive artery disease' OR 'peripheral occlusive arterial disease'/exp OR 'peripheral occlusive arterial disease' OR 'peripheral occlusive artery disease'/exp OR 'peripheral occlusive artery disease' OR 'peripheral occlusive disease'/exp OR 'peripheral occlusive disease' OR 'peripheral vascular occlusive disease'/exp OR 'peripheral vascular occlusive disease' OR 'peripheral arterial disease'/exp OR 'peripheral arterial disease'	91,702
	#2	'coronary disease'/exp OR 'coronary disease' OR 'multivessel coronary artery disease'/exp OR 'multivessel coronary artery disease' OR 'coronary artery disease'/exp OR 'coronary artery disease' OR 'coronary artery insufficiency'/exp OR 'coronary artery insufficiency' OR 'coronary heart disease'/exp OR 'coronary heart disease' OR 'coronary insufficiency'/exp OR 'coronary insufficiency' OR 'heart disease, coronary'/exp OR 'heart disease, coronary' OR 'heart disease, ischaemic'/exp OR 'heart disease, ischaemic' OR 'heart disease, ischemic'/exp OR 'heart disease, ischemic' OR 'ischaemia heart disease'/exp OR 'ischaemia heart disease' OR 'ischaemic cardiac disease'/exp OR 'ischaemic cardiac disease' OR 'ischaemic cardial disease'/exp OR 'ischaemic cardial disease' OR 'ischaemic cardiopathy'/exp OR 'ischaemic cardiopathy' OR 'ischaemic heart disease'/exp OR 'ischaemic heart disease' OR 'ischemia heart disease'/exp OR 'ischemia heart disease' OR 'ischemic cardiac disease'/exp OR 'ischemic cardiac disease' OR 'ischemic cardial disease'/exp OR 'ischemic cardial disease' OR 'ischemic cardiopathy'/exp OR 'ischemic cardiopathy' OR 'ischemic heart disease'/exp OR 'ischemic heart disease'	1,102,026
	#3	'ischaemic stroke'/exp OR 'ischaemic stroke' OR 'ischemic stroke'/exp OR 'ischemic stroke'	150,428
	#4	'angiocardiopathy'/exp OR 'angiocardiopathy' OR 'angiocardiovascular disease'/exp OR 'angiocardiovascular disease' OR 'cardiovascular complication'/exp OR 'cardiovascular complication' OR 'cardiovascular	6,034,547

		diseases'/exp OR 'cardiovascular diseases' OR 'cardiovascular disorder'/exp OR 'cardiovascular disorder' OR 'cardiovascular disturbance'/exp OR 'cardiovascular disturbance' OR 'cardiovascular lesion'/exp OR 'cardiovascular lesion' OR 'cardiovascular syndrome'/exp OR 'cardiovascular syndrome' OR 'cardiovascular vegetative disorder'/exp OR 'cardiovascular vegetative disorder' OR 'complication, cardiovascular'/exp OR 'complication, cardiovascular' OR 'disease, cardiovascular'/exp OR 'disease, cardiovascular' OR 'major adverse cardiovascular event'/exp OR 'major adverse cardiovascular event' OR 'cardiovascular disease'/exp OR 'cardiovascular disease'	
	#5	'estimated glucose disposal rate'/exp OR 'estimated glucose disposal rate' OR 'egdr'	389
	#6	#1 OR #2 OR #3 OR #4	6,057,812
	#7	#5 AND #6	258
Web of Science	#1	"TS=(Coronary Artery Disease OR Coronary Artery Diseases OR Coronary Arteriosclerosis OR Coronary Arterioscleroses OR Coronary Atheroscleroses OR Coronary Atherosclerosis OR Left Main Coronary Artery Disease OR Left Main Coronary Disease OR Left Main Disease OR Left Main Diseases OR Ischemic Heart Disease OR CAD OR CHD OR IDH) and Preprint Citation Index	906,673
	#2	"TS=(Ischemic Stroke OR Ischemic Strokes OR Ischaemic Stroke OR Ischaemic Strokes OR Acute Ischemic Stroke OR Acute Ischemic Strokes OR Cryptogenic Ischemic Stroke OR Cryptogenic Ischemic Strokes OR Cryptogenic Embolism Stroke OR Cryptogenic Embolism Strokes OR Cryptogenic Stroke OR Cryptogenic Strokes OR Wake-up Stroke OR Wake up Stroke OR Wake-up Strokes OR IS) and Preprint Citation Index	111,271,038
	#3	"TS=(Peripheral Arterial Disease OR Peripheral Arterial Diseases OR Peripheral Artery Disease OR PAD OR Peripheral Artery Diseases) and Preprint Citation Index	1,389,051
	#4	"TS=(Cardiovascular Diseases OR Cardiovascular Disease OR Cardiac Events OR Adverse Cardiac Event OR Adverse Cardiac Events OR Major Adverse Cardiac Events OR Atherosclerotic Cardiovascular Diseases OR CVD OR ASCVD) and Preprint Citation Index	3,114,509
	#5	"TS=(Estimated Glucose Disposal Rate OR eGDR) and Preprint Citation Index	733
	#6	"#1 OR #2 OR #3 OR #4 and Preprint Citation Index	112,859,242
	#7	"#5 AND #6 and Preprint Citation Index	544
Pubmed	#1	Coronary Artery Disease[Mesh] OR Artery Disease, Coronary OR Artery Diseases, Coronary OR Coronary Artery Diseases OR Coronary Arteriosclerosis OR Arterioscleroses, Coronary OR Coronary Arterioscleroses OR Arteriosclerosis, Coronary OR Atherosclerosis, Coronary OR Atheroscleroses, Coronary OR Coronary Atheroscleroses OR Coronary Atherosclerosis OR Left Main Coronary Artery Disease OR Left Main Coronary Disease OR Left Main Disease OR Left Main Diseases OR Ischemic Heart Disease	623,211
	#2	Ischemic Stroke[MeSH] OR Ischemic Strokes OR Stroke, Ischemic OR Ischaemic Stroke OR Ischaemic Strokes OR Stroke, Ischaemic OR Acute Ischemic Stroke OR Acute Ischemic Strokes OR Ischemic Stroke, Acute OR Stroke, Acute Ischemic OR Cryptogenic Ischemic Stroke OR Cryptogenic Ischemic Strokes OR Ischemic Stroke, Cryptogenic OR Stroke, Cryptogenic Ischemic OR Cryptogenic Embolism Stroke OR Cryptogenic Embolism Strokes OR Embolism Stroke, Cryptogenic OR Stroke, Cryptogenic Embolism OR Cryptogenic Stroke OR Cryptogenic Strokes OR Stroke, Cryptogenic OR Wake-up Stroke OR Stroke, Wake-up OR Wake up Stroke OR Wake-up Strokes	131,619
	#3	Peripheral Arterial Disease[Mesh] OR Arterial Disease, Peripheral OR Arterial Diseases, Peripheral OR Disease, Peripheral Arterial OR Diseases, Peripheral Arterial OR Peripheral Arterial Diseases OR	60,660

		Peripheral Artery Disease OR Artery Disease, Peripheral OR Artery Diseases, Peripheral OR Disease, Peripheral Artery OR Diseases, Peripheral Artery OR Peripheral Artery Diseases	
	#4	Cardiovascular Diseases[Mesh] OR Cardiovascular Disease OR Disease, Cardiovascular OR Cardiac Events OR Cardiac Event OR Event, Cardiac OR Adverse Cardiac Event OR Adverse Cardiac Events OR Cardiac Event, Adverse OR Cardiac Events, Adverse OR Major Adverse Cardiac Events OR Atherosclerotic Cardiovascular Diseases	3,138,191
	#5	Estimated Glucose Disposal Rate OR eGDR	429
	#6	((((Coronary Artery Disease[Mesh] OR Artery Disease, Coronary OR Artery Diseases, Coronary OR Coronary Artery Diseases OR Coronary Arteriosclerosis OR Arterioscleroses, Coronary OR Coronary Arterioscleroses OR Arteriosclerosis, Coronary OR Atherosclerosis, Coronary OR Atheroscleroses, Coronary OR Coronary Atheroscleroses OR Coronary Atherosclerosis OR Left Main Coronary Artery Disease OR Left Main Coronary Disease OR Left Main Disease OR Left Main Diseases OR Ischemic Heart Disease) OR (Ischemic Stroke[MeSH] OR Ischemic Strokes OR Stroke, Ischemic OR Ischaemic Stroke OR Ischaemic Strokes OR Stroke, Ischaemic OR Acute Ischemic Stroke OR Acute Ischemic Strokes OR Ischemic Stroke, Acute OR Stroke, Acute Ischemic OR Cryptogenic Ischemic Stroke OR Cryptogenic Ischemic Strokes OR Ischemic Stroke, Cryptogenic OR Stroke, Cryptogenic Ischemic OR Cryptogenic Embolism Stroke OR Cryptogenic Embolism Strokes OR Embolism Stroke, Cryptogenic OR Stroke, Cryptogenic Embolism OR Cryptogenic Stroke OR Cryptogenic Strokes OR Stroke, Cryptogenic OR Wake-up Stroke OR Stroke, Wake-up OR Wake up Stroke OR Wake-up Strokes)) OR (Peripheral Arterial Disease[Mesh] OR Arterial Disease, Peripheral OR Arterial Diseases, Peripheral OR Disease, Peripheral Arterial OR Diseases, Peripheral Arterial OR Peripheral Arterial Diseases OR Peripheral Artery Disease OR Artery Disease, Peripheral OR Artery Diseases, Peripheral OR Disease, Peripheral Artery OR Diseases, Peripheral Artery OR Peripheral Artery Diseases)) OR (Cardiovascular Diseases[Mesh] OR Cardiovascular Disease OR Disease, Cardiovascular OR Cardiac Events OR Cardiac Event OR Event, Cardiac OR Adverse Cardiac Event OR Adverse Cardiac Events OR Cardiac Event, Adverse OR Cardiac Events, Adverse OR Major Adverse Cardiac Events OR Atherosclerotic Cardiovascular Diseases)) AND (Estimated Glucose Disposal Rate OR eGDR)	140

Table 2: Detailed description of literature search strategy

Studies excluded	Reasons
Kong,2024 [1]	Cross-sectional study
Han,2024 [2]	Cross-sectional study
Xuan,2022 [3]	Cross-sectional study
Kim,2024 [4]	Insufficient data
Liao,2025 [5]	Cross-sectional study
Li,2024[6]	Insufficient data

Mutter, 2024[7]	Not target exposure: Different calculation methods of eGDR
Zheng,2024 [8]	Not target exposure: Changes in the eGDR
Penno,2021[9]	Not target outcome: all-cause mortality
Helmink,2021[10]	Not target exposure: Different calculation methods of eGDR
Zhang,2025[11]	Not target exposure: cumulative eGDR
Yan,2025[12]	Not target exposure: Changes in the eGDR
Yao,2024[13]	Not target exposure: eGDR control level
Kim,2025[14]	Not target exposure: Different calculation methods of eGDR
Yao,2024[15]	Not target exposure: eGDR control level
Liu,2023[16]	Not target outcome: Non-ST-Segment Elevation Acute Coronary Syndromes Undergoing Percutaneous Coronary Intervention
Liu,2022[17]	Not target outcome: non-ST-segment elevation acute coronary syndrome and non-diabetic patients after percutaneous coronary intervention
Zhu,2025[18]	Not target outcome: all-cause and cardiovascular mortality
Lu,2023[19]	Not target outcome: outcomes in acute ischemic stroke patients
Chen,2025[20]	Not target outcome: mortality risks<br>micro-and macrovascular complications
Karamanakos,2022[21]	Not target outcome: micro-and macrovascular

	complications
Chen,2025[22]	Not target outcome: mortality risk
He,2024[23]	Not target outcome: long-term mortality among individuals with and without diabetes
Song,2024[24]	Not target outcome: arterial stiffness and mortality
Guo,2024[25]	Not target outcome: cardiovascular mortality
Nyström,2017[26]	Not target outcome: long-term survival in type 2 diabetes after coronary artery bypass grafting
Feng,2024[27]	Not target exposure: eGDR with Neutrophil-to Lymphocyte Ratio Integrated
Hezaveh,2025[28]	Cross-sectional study
Olson,2022[29]	Not target outcome: mortality
Zooravar,2025[30]	Systematic review and meta-analysis
Sun,2025[31]	Systematic review and meta-analysis

1. Xiufang Kong, Wei Wang; Estimated glucose disposal rate and risk of cardiovascular disease and mortality in U.S. adults with prediabetes: a nationwide cross-sectional and prospective cohort study Acta diabetologica 2024 Nov;61(11):1413-1421 doi:10.1007/s00592-024-02305-1

2. Yutong Han, Kexin Zhang, Yue Luo, Bin Wan, Yaowen Zhang, Qinchuan Huang, Hanyu Liu, Yulin Leng, Chunguang Xie; Relationship between stroke and estimated glucose disposal rate: results from two prospective cohort studiesLipids in health and disease 2024 Nov 28;23(1):392 doi:10.1186/s12944-024-02385-6

3. Jin Xuan, Du Juan, Niu Yuyu, Ji Anjing; Impact of estimated glucose disposal rate for identifying prevalent ischemic heart disease: findings from a cross-sectional studyBMC cardiovascular disorders 2022 08 20;22(1):378 doi:10.1186/s12872-022-02817-0

4. Myung Jin Kim, Yun Kyung Cho, Eun Hee Kim, Min Jung Lee, Woo Je Lee, Hong-Kyu Kim, Chang Hee Jung; Association between estimated glucose disposal rate and subclinical coronary atherosclerosisNutrition, metabolism, and cardiovascular diseases : NMCD 2025 Jan;35(1):103686 doi:10.1016/j.numecd.2024.07.004

5. Jinhao Liao, Linjie Wang, Lian Duan, Fengying Gong, Huijuan Zhu, Hui Pan, Hongbo Yang; Association between estimated glucose disposal rate and cardiovascular diseases in patients with diabetes or prediabetes: a cross-sectional studyCardiovascular diabetology 2025 Jan 13;24(1):13 doi:10.1186/s12933-024-02570-y

6. Yan Li, Huijuan Li, Xiaoyu Chen, Xueyan Liang; Association between various insulin resistance indices and cardiovascular disease in middle-aged and elderly individuals: evidence from two prospectives nationwide cohort surveysFrontiers in

endocrinology 2024;15:1483468 doi:10.3389/fendo.2024.1483468

7. Stefan Mutter, Erika B Parente, Andrzej S Januszewski, Johan R Simonsen, Valma Harjutsalo, Per-Henrik Groop, Alicia J Jenkins, Lena M Thorn, FinnDiane Study Group; Insulin sensitivity estimates and their longitudinal association with coronary artery disease in type 1 diabetes. Does it matter?Cardiovascular diabetology 2024 May 03;23(1):152 doi:10.1186/s12933-024-02234-x eGDR 计算方式不同

8. Xiaowei Zheng, Wenyang Han, Yiqun Li, Minglan Jiang, Xiao Ren, Pinni Yang, Yiming Jia, Lulu Sun, Ruirui Wang, Mengyao Shi, Zhengbao Zhu, Yonghong Zhang; Changes in the estimated glucose disposal rate and incident cardiovascular disease: two large prospective cohorts in Europe and AsiaCardiovascular diabetology 2024 Nov 07;23(1):403 doi:10.1186/s12933-024-02485-8

9. Giuseppe Penno, Anna Solini, Emanuela Orsi, Enzo Bonora, Cecilia Fondelli, Roberto Trevisan, Monica Vedovato, Franco Cavalot, Gianpaolo Zerbini, Olga Lamacchia, Antonio Nicolucci, Giuseppe Pugliese, Renal Insufficiency And Cardiovascular Events (RIACE) Study Group; Insulin resistance, diabetic kidney disease, and all-cause mortality in individuals with type 2 diabetes: a prospective cohort studyBMC medicine 2021 03 15;19(1):66 doi:10.1186/s12916-021-01936-3

10. Marga A G Helmink, Marieke de Vries, Frank L J Visseren, Wendela L de Ranitz, Harold W de Valk, Jan Westerink; Insulin resistance and risk of vascular events, interventions and mortality in type 1 diabetesEuropean journal of endocrinology 2021 Nov 10;185(6):831-840 doi:10.1530/EJE-21-0636

11. Jin Zhang, Ziyi Sun, Yufei Li, Yuhan Yang, Wenjie Liu, Mengwen Huang, Kuiwu Yao; Association between the cumulative estimated glucose disposal rate and incident cardiovascular disease in individuals over the age of 50 years and without diabetes: data from two large cohorts in China and the United StatesCardiovascular diabetology 2025 Jan 31;24(1):51 doi:10.1186/s12933-025-02575-1

12. Lunqing Yan, Zhe Zhou, Xing Wu, Yumin Qiu, Zhefu Liu, Lifang Luo, Yang Yang, Xi Lu, Jiang He, Wenhao Xia; Association between the changes in the estimated glucose disposal rate and new-onset cardiovascular disease in middle-aged and elderly individuals: A nationwide prospective cohort study in ChinaDiabetes, obesity & metabolism 2025 Apr;27(4):1859-1867 doi:10.1111/dom.16179

13. Jiangnan Yao, Feng Zhou, Lingzhi Ruan, Yiling Liang, Qianrong Zheng, Jiaxin Shao, Fuman Cai, Jianghua Zhou, Hao Zhou; Association between estimated glucose disposal rate control level and stroke incidence in middle-aged and elderly adultsJournal of diabetes 2024 Aug;16(8):e13595 doi:10.1111/1753-0407.13595

14. Myung Jin Kim, Yun Kyung Cho, Eun Hee Kim, Min Jung Lee, Woo Je Lee, Hong-Kyu Kim, Chang Hee Jung; Association between estimated glucose disposal rate and subclinical coronary atherosclerosisNutrition, metabolism, and cardiovascular diseases : NMCD 2025 Jan;35(1):103686 doi:10.1016/j.numecd.2024.07.004

15. Jiangnan Yao, Feng Zhou, Lingzhi Ruan, Yiling Liang, Qianrong Zheng, Jiaxin Shao, Fuman Cai, Jianghua Zhou, Hao Zhou; Association between estimated glucose disposal rate control level and stroke incidence in middle-aged and elderly adultsJournal of diabetes 2024 Aug;16(8):e13595 doi:10.1111/1753-0407.13595

16. Chi Liu, Qi Zhao, Xiaoteng Ma, Yujing Cheng, Yan Sun, Dai Zhang, Yujie Zhou, Xiaoli Liu; Prognostic Value of Estimated Glucose Disposal Rate in Patients with Non-ST-Segment Elevation Acute Coronary Syndromes Undergoing Percutaneous Coronary InterventionReviews in cardiovascular medicine 2023 Jan;24(1):2 doi:10.31083/j.rcm2401002

17. Chi Liu, Xiaoli Liu, Xiaoteng Ma, Yujing Cheng, Yan Sun, Dai Zhang, Qi Zhao, Yujie Zhou; Predictive worth of estimated glucose disposal rate: evaluation in patients with non-ST-segment elevation acute coronary syndrome and non-diabetic patients after percutaneous coronary interventionDiabetology & metabolic syndrome 2022 Oct 06;14(1):145 doi:10.1186/s13098-022-00915-9

18. Botao Zhu, Chenghui Cao, Wenwu Liu, Yuxuan Liu, Yonghong Luo, Daoquan Peng; The predictive value of estimated glucose disposal rate for all-cause and cardiovascular mortality in the US non-diabetic population aged ≥60 years: A population-based cohort studyDiabetes & metabolic syndrome 2025 Jan;19(1):103182 doi:10.1016/j.dsx.2024.103182

19. Zhengzhao Lu, Yunyun Xiong, Xueyan Feng, Kaixuan Yang, Hongqiu Gu, Xingquan Zhao, Xia Meng, Yongjun Wang; Insulin resistance estimated by estimated glucose disposal rate predicts outcomes in acute ischemic stroke patientsCardiovascular diabetology 2023 08 26;22(1):225 doi:10.1186/s12933-023-01925-1

20. Xiaoli Chen, Aihua Li, Qilin Ma; Association of estimated glucose disposal rate with metabolic syndrome prevalence and mortality risks: a population-based studyCardiovascular diabetology 2025 Jan 22;24(1):38 doi:10.1186/s12933-025-02599-7

21. Georgios Karamanakos, Aikaterini Barmpagianni, Christos J Kapelios, Aikaterini Kountouri, Maria Bonou, Konstantinos Makrilakis, Vaia Lambadiari, John Barbetseas, Stavros Liatis; The association of insulin resistance measured through the

estimated glucose disposal rate with predictors of micro-and macrovascular complications in patients with type 1 diabetesPrimary care diabetes 2022 12;16(6):837-843 doi:10.1016/j.pcd.2022.10.003

22. Xiaoli Chen, Aihua Li, Qilin Ma; Association of estimated glucose disposal rate with metabolic syndrome prevalence and mortality risks: a population-based studyCardiovascular diabetology 2025 Jan 22;24(1):38 doi:10.1186/s12933-025-02599-7

23. Hao-Ming He, Ying-Ying Xie, Qiang Chen, Yi-Ke Li, Xue-Xi Li, Ya-Kun Mu, Xiao-Yan Duo, Yan-Xiang Gao, Jin-Gang Zheng; The additive effect of the triglyceride-glucose index and estimated glucose disposal rate on long-term mortality among individuals with and without diabetes: a population-based studyCardiovascular diabetology 2024 08 22;23(1):307 doi:10.1186/s12933-024-02396-8

24. Junting Song, Ruicong Ma, Lin Yin; Associations between estimated glucose disposal rate and arterial stiffness and mortality among US adults with non-alcoholic fatty liver disease Frontiers in endocrinology 2024;15:1398265 doi:10.3389/fendo.2024.1398265

25. Rubing Guo, Jingjing Tong, Yongtong Cao, Wei Zhao; Association between estimated glucose disposal rate and cardiovascular mortality across the spectrum of glucose tolerance in the US populationDiabetes, obesity & metabolism 2024 Dec;26(12):5827-5835 doi:10.1111/dom.15954

26. Thomas Nyström, Martin J Holzmann, Björn Eliasson, Ann-Marie Svensson, Jeanette Kuhl, Ulrik Sartipy; Estimated glucose disposal rate and long-term survival in type 2 diabetes after coronary artery bypass graftingHeart and vessels 2017 Mar;32(3):269-278 doi:10.1007/s00380-016-0875-1

27. Xunxun Feng, Yang Liu, Jiaqi Yang, Zhiming Zhou, Shiwei Yang, Yujie Zhou, Qianyun Guo; Evaluation of Estimated Glucose Disposal Rate with Neutrophil-to-Lymphocyte Ratio Integrated for Prognosticating Adverse Cardiovascular and Cerebrovascular Events and Risk Stratification Among Acute Coronary Syndrome with Type 2 Diabetes Mellitus Following Percutaneous Coronary InterventionJournal of inflammation research 2024;17:9193-9214 doi:10.2147/JIR.S490790

28. Ehsan Bahrami Hezaveh, Rana Hashemi, Mohammadamin Noorafrooz, Fatemeh Mohammadi, Amirhossein Yadegar, Sahar Karimpour Reyhan, Manouchehr Nakhjavani, Alireza Esteghamati, Soghra Rabizadeh; Estimated Glucose Disposal Rate: A Potential Determinant for Microvascular and Macrovascular Complications in Type 2 DiabetesEndocrinology, diabetes & metabolism 2025 Mar;8(2):e70037 doi:10.1002/edm2.70037

29. Jon C Olson, John R Erbey, Katherine V Williams, Dorothy J Becker, Daniel Edmundowicz, Sheryl F Kelsey, Kim Sutton Tyrrell, Trevor J Orchard; Subclinical atherosclerosis and estimated glucose disposal rate as predictors of mortality in type 1 diabetesAnnals of epidemiology 2002 Jul;12(5):331-7 doi:10.1016/s1047-2797(01)00269-1

30. Diar Zooravar, Hanieh Radkhah, Bahareh Shateri Amiri, Pedram Soltani; Estimated glucose disposal rate and microvascular complications of diabetes mellitus type I: A systematic review and meta-analysisDiabetes & vascular disease research 2025 Mar-Apr;22(2):14791641251324612 doi:10.1177/14791641251324612

31. Rui Sun, Jianxin Wang, Meng Li, Jingen Li, Yi Pan, Birong Liu, Gregory Y H Lip, Lijing Zhang; Association of Insulin Resistance With Cardiovascular Disease and All-Cause Mortality in Type 1 Diabetes: Systematic Review and Meta-analysisDiabetes care 2024 Dec 01;47(12):2266-2274 doi:10.2337/dc24-0475”

Table 3 Characteristics of the included cohort studies

**Author, Years, country**	**Study design/ Mean follow-up time**	**Study population**	**Sample sizes**	**Mean age (years)/Male (%)**	**Diabetes and prediabetes (%)/**	**Endpoint detection**	**Endpoint**	**eGDR analysis**	**Hazard risk (95% Cl)**	**Variables adjusted**
**Huan,2025, England [24]**	Prospective cohort study/13.8 years	UK Biobank without ASCVDs	360,953	56.3 ± 8.1 year/ 162 903 (45.1)	18 916 (5.2)	ICD-10	Myocardial infarction	Q1,<br>Q2,<br>Q3,	1.0(Ref),<br>0.90 (0.85–0.94),<br>0.78 (0.71–0.87),<br>0.59 (0.51–0.67),<br>0.88 (0.85–0.90)	Age, sex, race, smoking status, drinking status, index of multiple deprivation, sleep duration, sedentary time and healthy diet score, body mass index, family history of heart disease, hypertension, diabetes, low
							Ischemic stroke	Q4,<br>per 1-unit	1.0(Ref),<br>0.96 (0.90–1.04),	

									0.88 (0.77–1.00), 0.73 (0.61–0.89), 0.87 (0.83–0.90)	density lipoprotein cholesterol, c-reactive protein and serum creatinine
**Yi,2024, United States of America [25]**	Prospective cohort study/ 14.1 years	Six U.S. communities free of CVD	6,026	63.6±10.1/2863(47.5)	1711(28.4)	Trained personnel abstracted medical records suggestingpossible cardiovascular events.	ASCVD	4.3±1.2<br>6.6±0.6<br>9.0±0.5<br>10.7±0.6<br>per 1-unit	1 (Reference), 0.87 (0.68–1.10), 0.63 (0.47–0.84), 0.43 (0.30–0.64), 0.70 (0.60–0.80)	Age, gender, race, education, body mass index, and blood glucose status, low-density lipoprotein, high-density lipoprotein, triglyceride, and aspirin, angiotensin-converting enzyme inhibitors, angiotensin IIreceptor antagonists, β-blockers, statins, insulin, and oral hypoglycemic medications usage
							Myocardial infarction		1 (Reference), 0.73 (0.51–1.05), 0.60 (0.39–0.91), 0.37 (0.21–0.67) 0.72 (0.58–0.89)	
							Stroke		1 (Reference), 0.88 (0.60–1.27), 0.58 (0.37–0.92), 0.47 (0.26–0.85), 0.66 (0.52–0.82)	
**Zhang,2024,China [26]**	Prospective cohort study/6.62years	CHARLS cohort study without CVD	5,512	58.16±8.82/2983(54.1)	0	self-reported physician's diagnosis	CVD	6.58±0.69,<br>9.02±0.89,<br>10.76±0.27,<br>11.73±0.40,<br>per 1-unit	1 (Reference), 0.88 (0.76 −1.02) 0.69 (0.58 − 0.82) 0.66(0.56 − 0.79) 0.83 (0.78 −0.89)	Age, sex, rural residence, marital status, education, smoking, and alcohol consumption status, r region, TC, HDL, TG, LDL, BUN, UA, hsCRP, hemoglobin, chronic kidney disease, and obesity
							Stroke		1 (Reference), 0.69 (0.53−0.88), 0.52 (0.39−0.70), 0.42 (0.30−0.58), 0.70 (0.63−0.78)	
**Peng,2024, United States of America [27]**	Prospective cohort study/ 12.86 years	Prospective cohort study without CVD	19,906	59.41 ± 7.84/8,206(41.20)	0	ICD-10	ASCVD	<6.042,<br>6.042~7.731,<br>>7.731,<br>per 1-unit	1 (Reference), 0.892(0.807-0.987), 0.641(0.559-0.734), 0.868(0.844-0.893)	Age, sex, TDI, education, smoking, alcohol drinker status, ethnicity, eGFR, UACR, physical activity at goal, BMI, LDL-C, HDL-C, ALB, Hb, CRP, NLR, drugs aspirin, antihypertensive drug, lipid-lowering drugs,
							CHD		1(Reference), 0.859(0.766-0.962), 0.607(0.520-0.709), 0.858(0.832-0.886)	

							Stroke		1(Reference), 1.021(0.840-1.240), 0.748(0.579-0.966), 0.883(0.838-0.931)	
**Le, 2024, China [28]**	Prospective cohort study/7.25years	CHARLS cohort study without CVD	6,507	58.00 (52.00,64.00), 2,943 (45)	956 (15)	self-reported physician's diagnosis	CVD	6.29 (5.76, 6.71), 7.91 (7.49, 8.49), 10.47 (10.08, 10.77), 11.58 (11.30, 11.95)per 1-unit'	1(Reference), 0.76 (0.62, 0.93), 0.59 (0.48, 0.73), 0.50 (0.40, 0.63), 0.89 (0.86, 0.92)	Age, sex, local, marital, education, smoking, and drinking, HDL, BUN, UA, hs-CRP, Chronic kidney disease, BUN blood urea nitrogen
							Stroke		1(Reference), 0.95 (0.73, 1.23), 0.80 (0.61, 1.05), 0.65 (0.48, 0.87), 0.93 (0.89, 0.98)	
							CHD		1(Reference), 0.62 (0.47, 0.82), 0.40 (0.29, 0.56), 0.33 (0.23, 0.48), 0.82 (0.78, 0.87)	
**Ren,2022,China [29]**	Prospective cohort study/6 years	CHARLS cohort study without CVD	8,267	58.90 ± 9.40, 3916(43.4)	NA	self-reported physician's diagnosis	CVD	< 8.92, 8.92–10.41, 10.41–11.26, ⩾ 11.26 per 1-unit'	1(Reference), 0.95(0.83–1.10), 0.69(0.59–0.80), 0.58(0.49–0.67), 0.84(0.79–0.88)	age, sex, place of residence, education level, blood glucose, smoking, drinking, systolic blood pressure, physical activity, chronic diseases (dyslipidemia, chronic lung disease) and medications (anti-hypertensive and anti-dyslipidemic)
							Stroke		1(Reference), 1.01(0.81–1.27), 0.76(0.58–0.99), 0.70(0.53–0.94), 0.91(0.83–0.99)	
							Cardiac events		1(Reference), 0.94(0.81–1.09), 0.68(0.58–0.81), 0.57(0.47–0.68)	
**Zabala,2022, Swedish [30]**	Retrospective cohort study/5.6years	Swedish national diabetes without stroke	104, 697	62.9 ±11.5/ 58598(56.0%)	100%	ICD161-164	Stroke	< 4, 4-6,	1(Reference), 0.77 (0.69–0.84), 0.68 (0.58–0.80), 0.60 (0.48–0.76)	Age, sex, diabetes duration, LDL-C, HDL-C, tri-glycerides, lipid lowering medication, micro albuminuria, macroalbuminuria, creatinine, estimated glomerular filtration rate
							Ischaemic stroke		1(Reference), 0.75 (0.67–0.84),	

								6-8,<br>> 8	0.68 (0.57–0.81),<br>0.55 (0.43–0.71)	(eGFR), retinopathy, smoking, physical activity, disposable income, marital status, education, cardiovascular morbidities, renal disorder, hyperglycaemia, amputation, dementia, psychiatric disorder and gastric bypass surgery
**Liang,2025,China [31]**	Prospective study/ 8.29 ± 1.6	CHARLS cohort study without CVD	7849	59.00 ± 9.22/ 4146(52.82%)	1353 (17.24%)	self-reported physician's diagnosis	CVD	6.20 ± 0.79,<br>8.10 ± 0.66,<br>10.44 ± 0.41,<br>11.74 ± 0.70,<br>per 1-unit'	1(Reference),<br>0.73 (0.65, 0.82)<br>0.60 (0.53, 0.68)<br>0.50 (0.44, 0.58)<br>0.89 (0.87, 0.91)	age, gender, education level, marital status, living place, drinking status, smoking status, depression, WBC, platelets, BUN, FBG, Scr, TG, HDL-c, LDL-c, CRP, and UA
**Zhao,2025,China [32]**	Prospective study/ 9years	CHARLS cohort study without stroke	8,060	58.72 ± 8.92/ 3,647 (45.25%)	1,135 (14.08)	NA	Stroke	Q1,<br>Q2,<br>Q3,<br>Q4,<br>per 1-unit'	1(Reference),<br>0.74 (0.58,0.95),<br>0.65 (0.44,0.97),<br>0.52 (0.34,0.79),<br>0.62 (0.45,0.84)	eGDR, age, sex, location, marital_status, BMI, smoke, drink, hypertension, DM, dyslipidemia, heart.disease
**Li, 2024,China [33]**	Prospective study/9years	CHARLS cohort study without CVD	6426	57.9 ± 8.4/ 2906 (45.2%)	365 (4.2)	self-reported physician's diagnosis	CVD	Q1,<br>Q2,<br>Q3,<br>Q4,<br>per 1-unit'	1(Reference),<br>0.75 (0.61,0.91),<br>0.64 (0.51, 0.80),<br>0.59 (0.47, 0.74),<br>0.91 (0.88, 0.94)	Age, sex, marital status, education, smoking, and alcohol consumption status, region, total cholesterol, high density lipoprotein cholesterol, triglyceride, low density lipoprotein cholesterol, blood urea nitrogen, uric acid, hemoglobin, and obesity
							Stroke	Q1,<br>Q2,<br>Q3,<br>Q4,<br>per 1-unit'	1(Reference),<br>0.76 (0.55, 1.05),<br>0.73 (0.51, 1.04),<br>0.58 (0.39, 0.85),<br>0.91 (0.86, 0.97)	
**Tian,2025,China [34]**	Prospective study/7years	CHARLS cohort study without CVD	6725	58 (51, 64)<br>3,228 (48.00%)	4,010(59.63%)	self-reported physician's diagnosis	CVD	6.45 (5.91, 6.94,<br>8.48 (7.84, 9.49),<br>10.68 (10.39,10.92),<br>11.66 (11.39,11.99),<br>per 1-unit'	1(Reference),<br>0.81(0.68-0.96),<br>0.72(0.58-0.88),<br>0.74(0.58-0.94),<br>0.94(0.90-0.97)	Age sex, body mass index, huko, matal shatus, education levels, smoking shatus, dinking status diabetes, dyslipidema. depression, cancer, epprobems, SBP, DBP eGFR, TG, HDLC LDL-C, CRP,UA, and HGF
							Stroke		1(Reference),<br>0.91(0.74-1.12),<br>0.80(0.62-1.04),<br>0.71(0.52-0.97),<br>0.95(0.90-0.99)	
**Tan,2025, China [35]**	Prospective study/9years	CHARLS cohort study without CVD	6,359	59.61 ± 9.49/ 2,898 (45.57%)	262 (4.12%)	self-reported physician's diagnosis	CVD	≤ 6.966,<br>6.966 < eGDR ≤ 8.695,	1(Reference),<br>0.73(0.64,0.83),<br>0.65(0.56,0.76),	Gender, age, residence, marital status, education level, smoking status, drinking status, diabetes,

8.695 < eGDR ≤ 10.753, > 10.753, per 1-unit'	0.56(0.47,0.66), 0.91 (0.88, 0.93)	dyslipidemia, diabetes medications, dyslipidemia medications, platelets, CRP, BUN, FBG, Scr, HDL-C, UA, BMI, SBP, and DBP

eGDR, estimated glucose disposal rate; BP, blood pressures; BMI, body mass index; FPG, fasting plasma glucose; HbA1c, hemoglobin A1c; LDL-C, low-density lipoprotein-cholesterol; HDL-C, high-density lipoprotein-cholesterol; AST, aspartate aminotransferase; ALT, alanine aminotransferase; GGT, gamma glutamyl transferase; hs-CRP, high-sensitivity C-reactive protein; HOMA-IR, Homeostatic Model Assessment for Insulin Resistance; CAC, coronary artery calcium; SBP, systolic blood pressure， DBP, diastolic blood pressure; CRP, C - reactive protein; BUN, blood urea nitrogen; FBG, fasting blood glucose; Scr, serum creatinine; UA, uric acid; HGF, hepatocyte growth factor; eGFR, estimated glomerular filtration rate; Hb, Hemoglobin; NLR, neutrophil - to - lymphocyte ratio; TC, total cholesterol; TG, triglycerides; CVD, cardiovascular disease; CHARLS, china health and retirement longitudinal study; CHD, coronary heart disease; ASCVD, atherosclerotic cardiovascular disease.

Table 4: Details of quality evaluation via the Newcastle-Ottawa Scale

References	Selection				Comparability	Outcome			Total
	Exposed cohort	Nonexposed cohort	Ascertainment of exposure	Outcome of interest		Assessment of outcome	Length of follow-up	Adequacy of follow-up	
Huan,2025 [24]	*	*	*	*	*	*	*	*	8
Yi,2024 [25]	*	*	*	*	*	*	*	*	8
Zhang,2024 [26]	*	*	*	*	*	*	*	*	8
Peng,2024 [27]	*	*	*	*	*	*	*	*	8
Le, 2024 [28]	*	*	*	*	*	*	*	*	8
Ren,2022 [29]	*	*	*	*	*	*	*	*	8
Zabala,2022 [30]	*	*	*	*	*	*	*	*	8
Liang,2025 [31]	*	*	*	*	*	*	*	*	8
Zhao,2025 [32]	*	*	*	*	*	*	*	*	8
Li, 2024 [33]	*	*	*	*	*	*	*		7
Tian,2025 [34]	*	*	*	*	*	*	*		7
Tan,2025 [35]	*	*	*	*	*	*	*		7

Table 5 Dose-response analysis results of CVD/CHD

	**CVD**
**eGDR(mg/kg/min)**	HR(95%CI)
**0**	1.89(1.61-2.20)
**2**	1.37(1.27-1.48)
**4**	1.00(1.00-1.00)
**6**	0.73(0.67-0.79)
**8**	0.54(0.47-0.62)
**10**	0.44(0.39-0.50)
	Stroke
**eGDR(mg/kg/min)**	HR(95%CI)
**0**	1.38(1.01-1.88)
**2**	1.17(1.01-1.37)
**4**	1.00(1.00-1.00)
**6**	0.73(0.67-0.99)
**8**	0.56(0.47-0.85)
**10**	0.45(0.39-0.69)

Fig 1 Sensitivity analysis and funnel plot for the publication bias. A Sensitivity analysis for the meta-analysis of the association between eGDR and CVD risk. B Funnel plot for the publication bias underlying the meta-analysis of the association between eGDR and CVD risk. C Sensitivity analysis for the meta-analysis of the association between eGDR and stroke risk. D Funnel plot for the publication bias underlying the meta-analysis of the association between eGDR and stroke risk. HR, hazard ratio; CI, confidence interval; CVD, cardiovascular disease; eGDR, estimated glucose disposal rate.

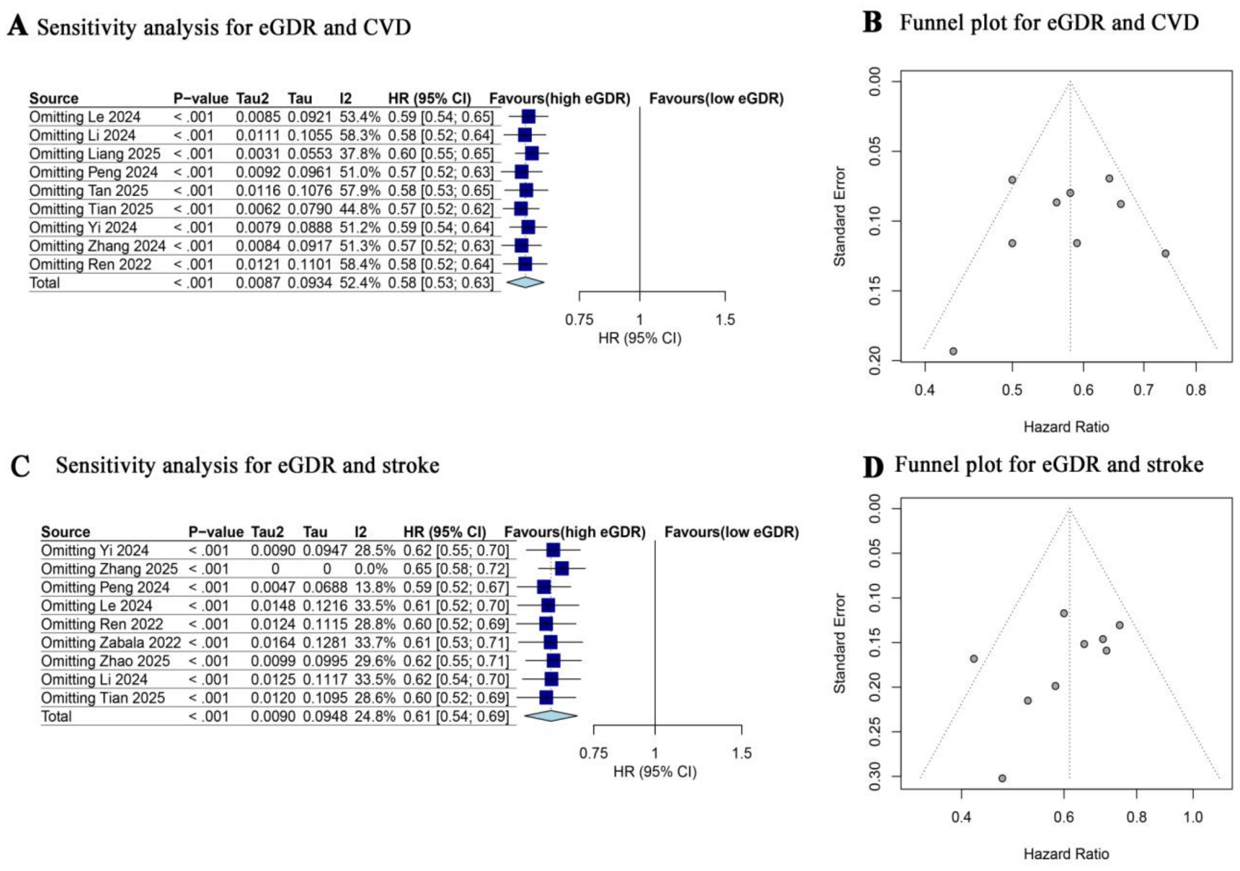

Fig 2 Begg's and Egger's regression for CVD and stroke. A Begg's regression for CVD. B Egger's regression for CVD. C Begg's regression for stroke. D Egger's regression for stroke. CVD, cardiovascular disease.

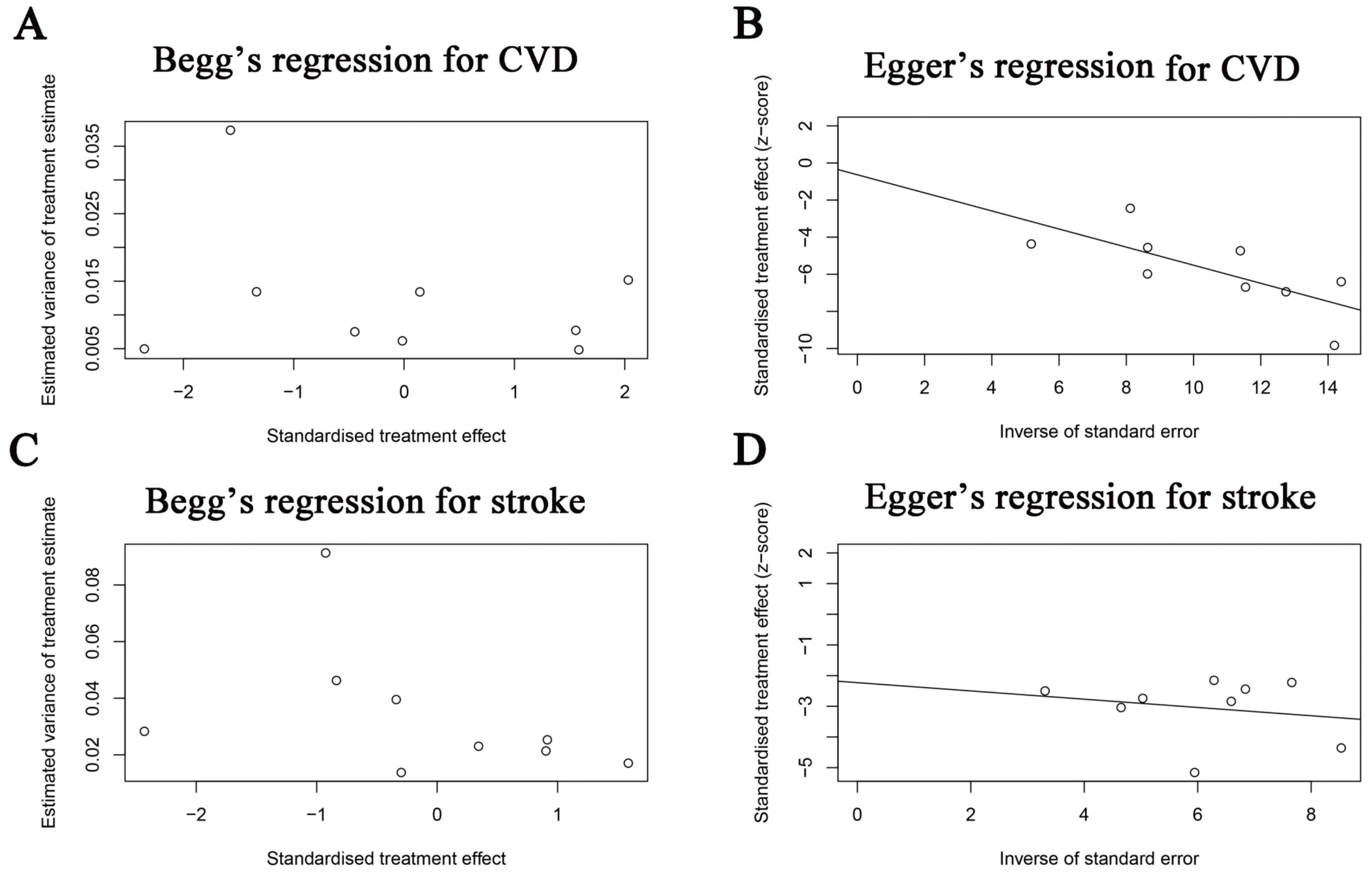

Fig 3 Trim-and-Fill Funnel Plot and Forest Plot After Trim-and-Fill for CVD and stroke. A Trim-and-Fill Funnel Plot for CVD. B Forest Plot After Trim-and-Fill for CVD. C Trim-and-Fill Funnel Plot for stroke. D Forest Plot After Trim-and-Fill for stroke. CVD, cardiovascular disease.

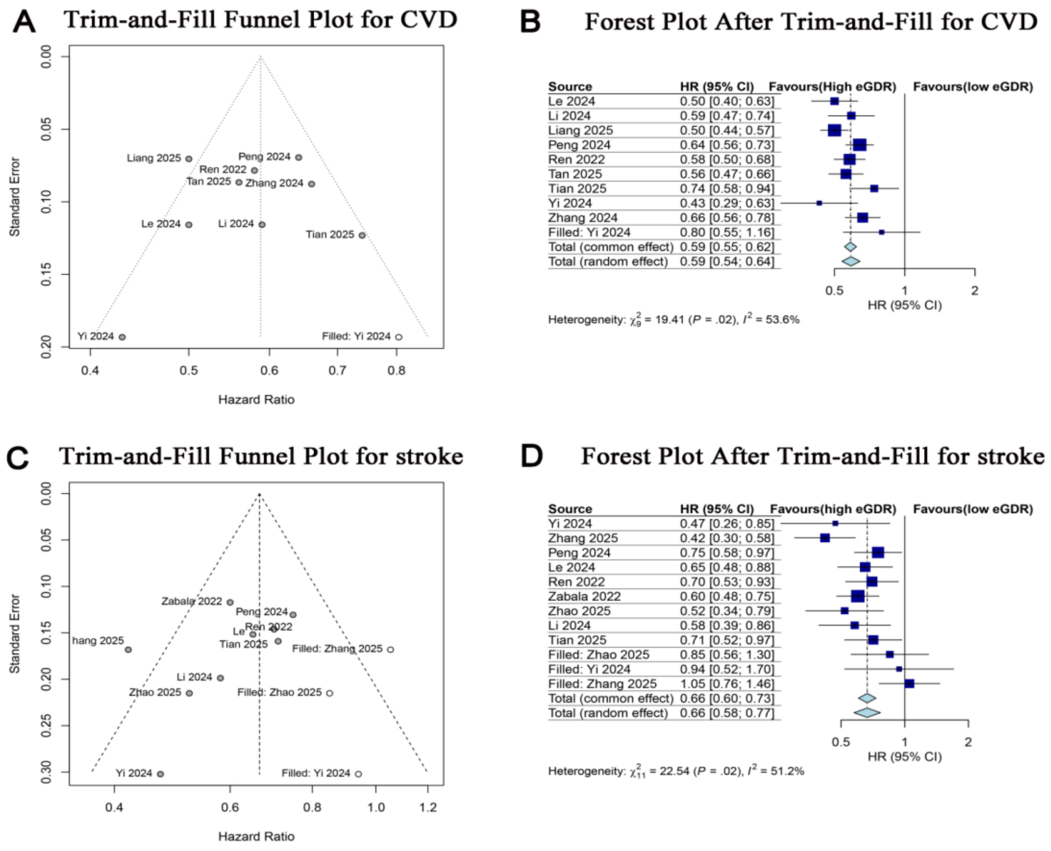

Supplement: Supplementary file 1 [file DataSheet1.pdf]
